# A systematic exploration of gut microbiota–driven blood metabolites in sepsis: an integrated bioinformatics and genetic association study

**DOI:** 10.3389/fgene.2026.1754817

**Published:** 2026-03-02

**Authors:** Yanhuo Zhang, Chenghao Qiu, Xingyu Li, Yaling Peng, Jun Liu, Peng Zhu

**Affiliations:** 1 Department of Gastrointestinal Surgery, The Second Affiliated Hospital of Chongqing Medical University, Chongqing, China; 2 The Second Clinical Medical College of Chongqing Medical University, Chongqing, China; 3 Department of Anesthesiology, Western Medicine Department, The Central Hospital of Enshi Tujia and Miao Autonomous Prefecture, Enshi, Hubei, China

**Keywords:** genome-wide association study, gut microbiota, metabolites, network pharmacology, sepsis

## Abstract

**Introduction:**

Alterations in the blood metabolome are closely associated with sepsis, while the gut microbiota (GM) plays a crucial role in modulating both sepsis progression and circulating metabolites. However, whether the effects of the GM on sepsis are mediated through blood metabolites remains unclear.

**Methods:**

To determine whether the effects of the GM on sepsis are mediated through blood metabolites, we performed a two-sample Mendelian randomization (MR) analysis combined with a two-step MR framework to identify potential metabolic mediators. Comprehensive bioinformatics analyses were integrated to construct interaction networks using Cytoscape, and pharmacodynamic experiments were conducted in a murine sepsis model.

**Results:**

We identified 23 GM taxa and 169 blood metabolites significantly associated with sepsis. Two-step MR analysis revealed that 15 metabolites mediated the causal relationships between 12 GM taxa and sepsis, with mediation proportions ranging from 3.70% to 13.70%. A total of 131 potential molecular targets were predicted for these metabolites, and network analysis highlighted five key metabolites and seven central targets. Molecular docking demonstrated strong binding affinities between these metabolites and their targets. Notably, gulonic acid (GA) and 4-hydroxyphenylacetic acid (4-HPA), driven by Lentisphaerae, Lentisphaeria, and Victivallales, significantly improved survival and attenuated organ injury and inflammation in septic mice.

**Discussion:**

Collectively, this study provides evidence supporting a causal role of the GM in sepsis, which mediated in part by blood metabolites. These findings highlight the therapeutic potential of targeting both the GM and GM-driven metabolites as novel interventions for sepsis.

## Graphical Abstract

**Figure:**
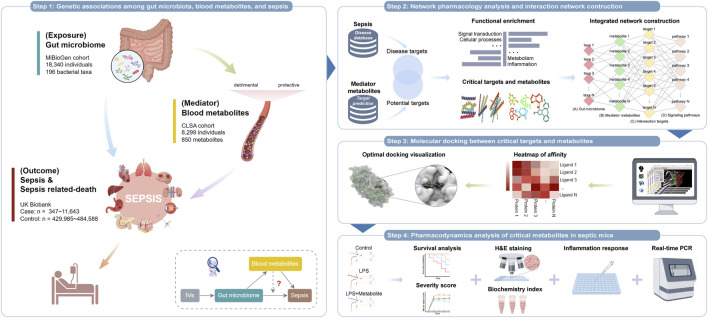

## 1 Introduction

Sepsis is a life-threatening syndrome triggered by diverse infections and characterized by a dysregulated host immune response, leading to multiple organ dysfunction, including the heart, lungs, kidneys, and liver. It frequently develops secondary to severe trauma, extensive burns, or major surgery, making it one of the leading causes of mortality among critically ill surgical patients (Angus and van der Poll, 2013). According to data from the Global Burden of Disease (GBD) 2017 study, sepsis accounted for approximately 19.7% of all deaths worldwide, imposing a substantial global healthcare burden (Rudd et al., 2020). Despite advances in critical care, anti-infective therapy and organ support remain the mainstay of treatment, with no other approved therapeutic options currently available (Marshall, 2014). This underscores the urgent need to develop effective preventive and therapeutic strategies. As an emerging environmental determinant of host physiological homeostasis (Kamada et al., 2013), the gut microbiota (GM) has been increasingly recognized as a pivotal contributor to the onset and progression of sepsis (Liu et al., 2019). Mounting evidence indicates a bidirectional relationship between GM dysbiosis and sepsis: disruption of the gut microbial community can predispose to sepsis, while sepsis itself exacerbates intestinal dysbiosis (Adelman et al., 2020; Haak and Wiersinga, 2017). Clinical studies have demonstrated that the abundance of beneficial commensals markedly decreases, whereas opportunistic pathogens expand during sepsis. For instance, pathogenic taxa such as *Enterococcus*, *Klebsiella*, *Clostridia*, and *Proteobacteria* are significantly enriched, while beneficial bacteria including *Blautia*, *Faecalibacterium*, *Prevotella*, *Firmicutes*, and *Lachnospiraceae* are notably depleted in septic patients (O'Keefe et al., 2011; Mu et al., 2022; Sun et al., 2023a; Rajilić-Stojanović and de Vos, 2014).

Blood circulates throughout the body, facilitating the transport of nutrients and metabolites among various organs. It is well recognized that the therapeutic effects observed after oral drug administration often result from changes in bioactive components within the bloodstream. Consequently, blood serves as an ideal entry point for investigating systemic metabolic processes. Alterations in the blood metabolome are closely linked to a wide range of human diseases, including cardiovascular disorders, neuropsychiatric conditions, autoimmune diseases (Wei et al., 2023), and sepsis (Chen Q. et al., 2022; Su et al., 2014; Jaurila et al., 2020). Intriguingly, a recent study demonstrated that host genetics and the GM collectively shape approximately 64% of circulating metabolites, with 69% driven solely by the GM, 15% by host genetics, and 16% by combined genetic-microbial influences, indicating that the GM is the predominant determinant of the blood metabolome (Diener et al., 2022). Consistent with this, a Mendelian randomization (MR) analysis supported a causal relationship between the GM and circulating metabolites (Liu et al., 2022). Furthermore, integrative multi-omics and MR analyses revealed that depletion of blood 3-indolepropionic acid and N-methyltryptamine, key microbial effectors derived from *Eubacterium rectale* and *Clostridium sp. CAG_299*, was causally associated with elevated non–high-density lipoprotein cholesterol (non-HDL-C), a recognized risk factor for cardiovascular disease (Zhou et al., 2024). More importantly, accumulating animal and clinical evidence indicates that the GM can modulate the progression and prognosis of sepsis by altering circulating levels of bioactive metabolites. These include PE (0:0/14:0) (a phosphatidylethanolamine) (Tian et al., 2023), Arg-Lys-His (a novel bioactive tripeptide, RKH) (Xie et al., 2023), hyodeoxycholic acid (Li et al., 2023), L-arginine (Nüse et al., 2023), indole 3-propionic acid (IPA) (Huang et al., 2022), and 5-hydroxyindoleacetic acid (Li et al., 2022). Despite growing evidence linking the GM, blood metabolites, and sepsis, the causal relationships among them remain unclear, largely due to confounding factors and reverse causality inherent in conventional observational studies. These limitations can be addressed using MR, an analytical approach that serves as a natural alternative to randomized controlled trials (RCTs) by employing genetic variants as instrumental variables (IVs) to infer causal relationships between exposures and outcomes (Smith and Ebrahim, 2003). Therefore, we performed MR analyses using summary statistics from large-scale genome-wide association studies (GWAS) of GM, blood metabolites, and sepsis to elucidate their causal interconnections and to identify specific GM taxa and metabolites potentially contributing to sepsis pathogenesis and therapeutic development. Furthermore, we implemented a mediation MR framework (two-step MR) to verify and quantify metabolic mediators that may bridge the causal associations between the GM and sepsis (Chen Z. et al., 2023).

On one hand, critically ill patients, including those with sepsis, have shown tangible therapeutic benefits from GM–based interventions. These primarily include fecal microbiota transplantation (FMT), selective digestive decontamination (SDD), supplementation with probiotics or prebiotics, and direct administration of GM-derived metabolites such as flavonoids (He et al., 2023). On the other hand, network pharmacology, originally developed for investigating traditional Chinese medicine, has proven effective in capturing the complex interactions between biomolecules and chemical constituents. It has been successfully applied to a range of human diseases to elucidate the pharmacological (Li et al., 2021) or toxicological (Huang, 2023) molecular targets and mechanisms of various bioactive compounds, including GM-related metabolites (Oh et al., 2022; Oh et al., 2023a; Oh et al., 2023b). Therefore, network pharmacology represents a powerful approach for uncovering the molecular targets and underlying mechanisms through which GM-derived mediator metabolites influence sepsis. In this study, we developed an integrative strategy combining MR, network pharmacology, and molecular docking to elucidate the intricate interaction network linking sepsis, GM, blood metabolites, molecular targets, and signaling pathways. Furthermore, we hypothesized that exogenous supplementation of specific metabolites might exert either protective or detrimental effects on sepsis outcomes. Based on the integrated analysis, we identified and prioritized novel GM-driven metabolites with the strongest potential to mitigate sepsis and subsequently validated their therapeutic effects in a murine model of sepsis. Collectively, our findings underscore the potential of targeting the GM and their associated metabolites as promising therapeutic avenues for the management of sepsis.

## 2 Materials and methods

### 2.1 Study design

This study comprised four major components, as illustrated in the graphical abstract. First (Step 1), a network MR analysis was performed to identify causal relationships between the GM and sepsis-related outcomes, as well as to determine potential GM-driven metabolic mediators. Second (Step 2), network pharmacology was applied to explore the underlying molecular targets and mechanisms of these mediator metabolites. Interaction networks were then constructed using Cytoscape to identify core metabolites and key targets. Third (Step 3), molecular docking was conducted to evaluate binding interactions between the core metabolites and their corresponding targets. Finally (Step 4), *in vivo* experiments were carried out in a murine sepsis model to validate the protective effects of specific metabolites. The MR analysis was conducted under the framework of three fundamental assumptions, which have been comprehensively described in previous studies (Bowden and Holmes, 2019). Additionally, the study adhered to the STROBE-MR (Strengthening the Reporting of Observational Studies in Epidemiology Using Mendelian Randomization) reporting guidelines, and the corresponding checklist was completed (Skrivankova et al., 2021).

### 2.2 Mendelian randomization analysis

#### 2.2.1 Data source

Summary statistics for the GM were obtained from the MiBioGen consortium, which included 18,340 participants across 24 cohorts, of whom approximately 78% were of European ancestry (Kurilshikov et al., 2021). The MiBioGen consortium curated and analyzed 16S rRNA gene sequencing data from fecal samples, yielding 196 taxonomic units comprising 119 genera, 32 families, 20 orders, 16 classes, and 9 phyla. Taxon abundances were represented as relative abundances. Summary-level data for plasma metabolites were derived from a large-scale GWAS involving 8,299 unrelated European participants enrolled in the Canadian Longitudinal Study of Aging (CLSA) (Chen Y. et al., 2023). This dataset included 850 identified metabolites and approximately 15.4 million single nucleotide polymorphisms (SNPs), classified into eight major metabolic super-pathways: lipids, amino acids, xenobiotics, nucleotides, cofactors and vitamins, carbohydrates, peptides, and energy metabolism. GWAS summary statistics for sepsis, sepsis requiring critical care, and 28-day mortality in both conditions were obtained from the United Kingdom Biobank consortium, with adjustments made for sex and age. MiBioGen GM GWAS summary statistics were downloaded directly from the MiBioGen portal as full summary files provided by taxonomic level (https://mibiogen.gcc.rug.nl/menu/main/home/). Plasma metabolite and sepsis GWAS summary statistics were obtained via the GWAS Catalog (https://www.ebi.ac.uk/gwas/downloads/summary-statistics), where the study deposited summary-level results with specific accession numbers; files can be retrieved by searching the accession in the GWAS Catalog “Summary statistics” download page and downloading the corresponding files from the Catalog’s FTP directory. In MiBioGen, mbQTL analyses were performed on covariate-adjusted taxon abundance after excluding samples with zero abundance; for plasma metabolites in CLSA, Metabolon batch-normalized data were used and only metabolites with <50% missing measurements were retained before transformation and GWAS. All participants in both the case and control groups were of European descent. More details were also provided (Bycroft et al., 2018).

#### 2.2.2 Selection of genetic instrumental variables

For the MR analysis, it is essential that the selected genetic IVs accurately represent the exposure traits. While a genome-wide significance threshold of *P* < 1 × 10^−8^ is commonly used, microbiome GWAS typically provide limited genome-wide significant variants for many taxa; therefore, SNPs associated with GM taxa and blood metabolites were selected using a suggestive threshold of *P* < 1 × 10^−5^, an approach widely adopted in gut microbiome MR studies to retain sufficient instruments (Lv et al., 2024; Wang et al., 2023). To ensure independence among variants, SNPs were pruned based on linkage disequilibrium (LD) using an *r* ^*2*^ < 0.1 within a 500 kb window (Chen Y. et al., 2024). When no shared SNPs were available between the exposure and outcome datasets, proxy SNPs (*r* ^*2*^ ≥ 0.8) were identified from the 1000 Genomes European reference panel. SNPs directly associated with the outcome (*P* < 1 × 10^−5^) and those with *F*-statistics <10 were excluded to minimize bias from weak instruments. To avoid unstable single-variant (Wald ratio) estimates and to enable pleiotropy/heterogeneity-robust sensitivity analyses that require multiple instruments, only GM taxa and metabolites represented by at least three valid SNPs were retained for downstream analyses. In addition, we required the instrument set to explain a minimum proportion of exposure variance (*R* ^*2*^ ≥ 0.5%) to reduce extremely low-power analyses, since MR power depends strongly on the variance explained by the genetic instruments (Burgess, 2014). Potential pleiotropic associations of selected SNPs were assessed using the PhenoScanner database (Kamat et al., 2019). The proportion of explained variance (*R* ^*2*^) and the strength of the instruments (*F*-statistic) were calculated according to the following formulas: *R* ^*2*^ = 2 × MAF × (1−MAF) × β^2^, *F* = *R* ^*2*^ × (n-k-1)/k × (1-*R* ^*2*^), where MAF denotes the minor allele frequency, β denotes the estimated per-allele effect size of the SNP on the exposure, n is the sample size, and k is the number of IVs included (Burgess et al., 2011).

#### 2.2.3 Two-sample mendelian randomization analysis

In this MR analysis, the inverse variance weighted (IVW) method served as the primary analytical approach. The IVW model assumes the absence of horizontal pleiotropy across all SNPs and integrates the Wald ratios of each genetic variant through an IVW meta-analysis (Pierce and Burgess, 2013). To assess the robustness of causal estimates, several sensitivity analyses were conducted. The MR-Egger intercept test and the Mendelian Randomization Pleiotropy RESidual Sum and Outlier (MR-PRESSO) global test were used to detect the presence of directional and horizontal pleiotropy. Heterogeneity among SNPs was evaluated using Cochran’s Q statistic; when heterogeneity was detected (*P* < 0.05), a random-effects IVW model was applied, whereas a fixed-effects IVW model was used when heterogeneity was absent (*P* > 0.05). To further confirm the consistency and reliability of causal inference, eight additional MR methods were employed, including Bayesian weighted Mendelian randomization (BWMR) (Zhao et al., 2020), maximum likelihood (Burgess et al., 2016), MR-Egger (Bowden et al., 2015), MR-PRESSO (Verbanck et al., 2018), simple median (Bowden et al., 2016), simple mode (Hartwig et al., 2017), weighted median (Bowden et al., 2016), and weighted mode (Hartwig et al., 2017) analyses. The Steiger directionality test was also performed to determine whether the causal estimates were influenced by reverse causation. A causal relationship was considered robust when the following criteria were met: (i) IVW-derived *P* < 0.05, (ii) consistent direction of effect across all MR methods, and (iii) no evidence of horizontal pleiotropy, as indicated by a non-significant MR-Egger intercept and MR-PRESSO global test. Bonferroni correction was used to account for multiple testing. For GM taxa, significance thresholds were set as 0.05 divided by the number of taxa tested within each taxonomic level (e.g., order: 0.05/20), and for plasma metabolites as 0.05/850. These corrections were applied separately for each sepsis outcome. All MR analyses were performed using the TwoSampleMR and MRPRESSO packages in R.

#### 2.2.4 Mediation mendelian randomization analysis

A two-step MR analysis was performed to identify metabolic mediators that potentially bridge the associations between the GM and various sepsis-related outcomes. Specifically, the causal effects of (i) exposure on mediator (denoted as β1), (ii) mediator on outcome (β2), and (iii) exposure on outcome (β0) were estimated using two-sample MR analyses. When β0, β1, and β2 were all statistically significant, a causal relationship between the exposure and outcome was inferred, with the possibility that the mediator partially accounted for this association (Chen Z. et al., 2023). The indirect (mediated) effect was calculated as β1×β2, and the proportion of mediation was determined as (β1×β2)/β0. Standard errors (SEs) for the mediated effects were estimated using the delta method (Tofighi and MacKinnon, 2011).

### 2.3 Network pharmacology analysis

#### 2.3.1 Construction of a sepsis-target-metabolite network

Sepsis-related targets were first identified using the keyword “sepsis” to query the DisGeNET (https://www.disgenet.org/), GeneCards (https://www.genecards.org/), Comparative Toxicogenomics Database (CTD, http://ctdbase.org/), and Therapeutic Target Database (TTD, http://db.idrblab.net/ttd/). In addition, five publicly available gene expression datasets, including GSE9960, GSE28750, GSE65682, GSE95233, and GSE134347, were downloaded from the Gene Expression Omnibus (GEO, https://www.ncbi.nlm.nih.gov/geo/) database, containing blood transcriptomic data from septic patients and healthy controls. The robust rank aggregation (RRA) method was employed to integrate differential expression results across platforms and to identify consistently and significantly altered genes as the final set of differentially expressed genes (DEGs) (Kolde et al., 2012). Targets that appeared in at least two of the aforementioned databases were retained as putative therapeutic targets for sepsis. Subsequently, the SMILES strings and 3D structures of the mediator metabolites were obtained from the PubChem and Human Metabolome Database (HMDB, https://hmdb.ca/). These structures were uploaded to Super-PRED (https://prediction.charite.de/) (Nickel et al., 2014), PharmMapper (http://www.lilab-ecust.cn/pharmmapper/) (Wang et al., 2017), SwissTargetPrediction (STP, http://www.swisstargetprediction.ch/) (Daina and Zoete, 2024), and TargetNet (http://targetnet.scbdd.com/) (Yao et al., 2016) for target prediction. After removing duplicates and merging the results, a comprehensive list of metabolite-related targets was generated. The intersection of sepsis-related targets and metabolite-related targets was defined as the set of putative targets through which mediator metabolites may exert their effects on sepsis. The sepsis-target-metabolite interaction network was then constructed using Cytoscape software (version 3.7.2). Finally, the cytoHubba plug-in within Cytoscape was applied to identify key metabolites based on the Degree algorithm.

#### 2.3.2 Construction and analysis of a protein-protein interaction (PPI) network

To investigate the putative targets at a systems level, protein-protein interaction (PPI) analysis was conducted using the STRING database (version 12.0; https://cn.string-db.org/). The putative targets were uploaded to STRING under the *Homo sapiens* setting with a high-confidence interaction threshold. Free (unconnected) proteins were excluded, and the resulting PPI network was visualized using Cytoscape software. Core targets within the network were identified using the cytoHubba plug-in of Cytoscape, while potential functional modules were detected using the MCODE plug-in. Gene Ontology (GO) enrichment analyses for each identified module were performed with the Hs.e.g.,.db and clusterProfiler R packages.

#### 2.3.3 Functional enrichment analysis

GO and Kyoto Encyclopedia of Genes and Genomes (KEGG) enrichment analyses of the putative targets were performed using the clusterProfiler R package. The GO analysis included three categories: biological process (BP), molecular function (MF), and cellular component (CC). Visualization of the enrichment results was carried out with the ggplot2 R package. KEGG pathway enrichment results were categorized and summarized according to the KEGG PATHWAY database. Pathways that were clearly unrelated to sepsis were excluded, and a target-pathway interaction network was subsequently constructed using Cytoscape software.

### 2.4 Molecular docking

Based on the above results, molecular docking was performed to evaluate the interactions between the critical metabolites and core target proteins. The 3D structures of the target proteins (CXCL8: 4XDX, IL6: 4J4L, MAPK1: 8AOJ, MAPK3: 4QTB, MMP1: 3SHI, MMP9: 6ESM, and STAT3: 6NJS) were retrieved in PDB format from the Protein Data Bank (PDB; https://www.rcsb.org/). Protein structures were preprocessed using PyMOL (version 2.5) and AutoDockTools (version 1.5.7) (Zhang and Sanner, 2019) by removing bound ligands and water molecules and adding hydrogen atoms. The 3D structures of the metabolites were obtained from the PubChem database. Processed protein structures were then converted from PDB to PDBQT format using AutoDockTools. Molecular docking was conducted with AutoDock Vina (version 1.1.2) (Eberhardt et al., 2021) and docking results were analyzed and visualized using the Protein-Ligand Interaction Profiler (PLIP) web tool (https://plip-tool.biotec.tu-dresden.de/plip-web/plip/index).

### 2.5 Experimental evaluation

#### 2.5.1 Animals and treatment

Male C57BL/6J mice (8 weeks old, 22 ± 2 g) were obtained from Vital River Laboratory Animal Technology (Beijing, China). All mice were housed under specific pathogen-free (SPF) conditions at the Experimental Animal Center of Chongqing Medical University (Chongqing, China). Animals were maintained in individually ventilated cages under a 12 h light/dark cycle, with controlled temperature (20 °C–25 °C) and humidity (50% ± 5%), and provided *ad libitum* access to standard chow and water. Lipopolysaccharide (LPS; Sigma-Aldrich, USA; L2630), gulonic acid (GA; GLPBIO, USA; GF11075), and 4-hydroxyphenylacetic acid (4-HPA; GLPBIO, USA; GC33815) were purchased from the respective suppliers. After 1 week of acclimatization, the mice were randomly assigned to six groups (n = 6 per group): control, LPS, LPS + GA, and LPS + 4-HPA groups.

To induce sepsis, mice were intraperitoneally injected with LPS at a dose of 10 mg/kg. GA (200 mg/kg) and 4-HPA (100 mg/kg) were administered intraperitoneally 60 min prior to LPS injection. After 24 h, the mice were euthanized with an overdose of sodium pentobarbital, and blood and multiple organ tissues were collected for histopathological and molecular analyses. For the survival study, a lethal LPS dose (20 mg/kg) was used (Dao et al., 2024). Each group contained 12 mice, and survival status was monitored every 12 h for 72 h. Sepsis severity was assessed every 12 h for 48 h using the murine sepsis score (MSS), which evaluates clinical parameters such as appearance and level of consciousness, as described by Shrum et al. (2014). All animal experiments conducted in this study followed the national ethical principles and standards for animal welfare and were approved by the Ethics Committee of the Second Affiliated Hospital of Chongqing Medical University.

#### 2.5.2 Blood biochemical parameters assays

Collected blood samples were centrifuged at 3,000 rpm for 15 min to obtain serum. The serum levels of lactate dehydrogenase (LDH), creatine kinase-MB isoenzyme (CK-MB), creatinine (Cr), blood urea nitrogen (BUN), alanine aminotransferase (ALT), and aspartate aminotransferase (AST) were measured using an automated biochemical analyzer (TBA-120FR; TOSHIBA, Japan). All assays were conducted in strict accordance with the manufacturer’s instructions.

#### 2.5.3 Hematoxylin and eosin staining

Liver, lung, and kidney tissues were fixed in 10% paraformaldehyde for at least 48 h, embedded in paraffin, and sectioned into 4-µm-thick slices. The paraffin sections were dewaxed with xylene and rehydrated through a graded ethanol series. Hematoxylin and eosin (H&E) staining was then performed, followed by dehydration, clearing, and mounting. Pathological changes were examined under an optical microscope (BX43; Olympus, Japan).

#### 2.5.4 Detection of inflammatory cytokines and oxidative stress biomarkers

Serum concentrations of inflammatory cytokines, including tumor necrosis factor-alpha (TNF-α), interleukin-6 (IL-6), IL-10, and IL-1β, were quantified using commercial enzyme-linked immunosorbent assay (ELISA) kits (Thermo Fisher Scientific, USA). In addition, serum levels of oxidative stress markers, including glutathione (GSH), superoxide dismutase (SOD), malondialdehyde (MDA), and catalase (CAT), were determined using commercial assay kits (Servicebio, China).

#### 2.5.5 Quantitative real-time PCR (RT-qPCR)

Total RNA of liver tissues was abstracted using TRIzol Reagent (Life Technologies, United States). The RNA was reverse-transcribed to cDNA utilizing All-In-One 5×RT MasterMix (abm, China, Cat.No.G592). RT-qPCR was performed on the CFX Connect™ Real-Time System (Bio-Rad, Hercules, CA, United States) using BlasTaq™ 2×qPCR MasterMix (abm, China, Cat.No.G891). The relative mRNA expression was calculated by the 2^−ΔΔCT^ method using β-actin as the internal reference. The primer sequences are listed in Table 1.

**TABLE 1:** Primers sequences for RT-qPCR.

Gene (mouse)	Sense primer (5′–3′)	Antisense primer (5′–3′)
β-actin	CAT​CCG​TAA​AGA​CCT​CTA​TGC​CAA​C	ATG​GAG​CCA​CCG​ATC​CAC​A
IL6	TGA​TGG​ATG​CTA​CCA​AAC​TGG​A	TGA​TGG​ATG​CTA​CCA​AAC​TGG​A
MMP1	GTG​CCT​GAT​GTG​GGT​GAA​TA	TGT​CAG​CAG​TGC​CAT​CAT​AG
MMP9	GCC​CTG​GAA​CTC​ACA​CGA​CA	TTG​GAA​ACT​CAC​ACG​CCA​GAA​G
MAPK1	TTG​CTT​TCT​CTC​CCG​CAC​AA	GGG​CTC​ATC​ACT​TGG​GTC​ATA
MAPK3	CAA​CAC​CAC​CTG​CGA​CCT​TA	GGA​TTT​GGT​GTA​GCC​CTT​GGA​A
STAT3	TCC​TGG​CAC​CTT​GGA​TTG​AG	TGT​GCT​GAT​AGA​GGA​CAT​TGG​A

### 2.6 Statistical analysis

Data are presented as the mean ± standard error of the mean (SEM). All statistical analyses were conducted using GraphPad Prism (version 8.0), SPSS (version 26.0), and R (version 4.3.1) software. Comparisons between two groups were performed using Student’s t-test, while differences among multiple groups were analyzed using one-way analysis of variance (ANOVA). Survival curves were plotted using the Kaplan-Meier method and compared with the log-rank test. A *P*-value <0.05 was considered statistically significant.

## 3 Result

### 3.1 Mendelian randomization analysis

#### 3.1.1 Genetic IVs included in analysis

A total of 2,126 SNPs were selected as IVs for the 196 GM taxa, with the number of IVs per taxon ranging from 4 to 26. For the 850 blood metabolites, 23,621 SNPs were identified as IVs, ranging from 12 to 174 per metabolite. All selected SNPs exhibited *F*-statistics greater than 10, ranging from 14.59 to 88.43 for GM and from 10.02 to 3,818.76 for metabolites, indicating no evidence of weak instruments. Detailed characteristics of the IVs are presented in Supplementary Tables S1, S2.

#### 3.1.2 Causal effects of gut microbiota on sepsis

**FIGURE 1:**
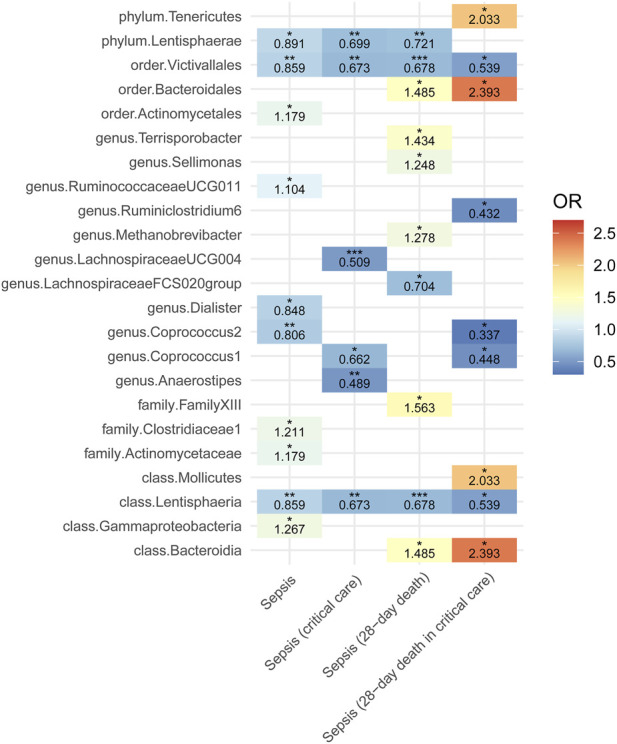
Heatmap showing the Mendelian randomization results between gut microbiota and sepsis and sepsis-related outcomes using inverse variance weighted method. OR, odds ratio. Red represents risk factor; blue represents protective factor. ^*^ *P* < 0.05, ^**^ *P* < 0.01, ^***^ *P* < 0.001.

A total of 23 GM taxa were identified as being causally associated with sepsis-related outcomes. Among these, one order, one class, one phylum, and seven genera were negatively associated with sepsis outcomes, whereas two orders, three classes, one phylum, three families, and four genera showed positive associations (Figure 1; Supplementary Table S3). Notably, the MR results remained stable even when two taxa were grouped as subcategories of the same phylum or another higher-level classification. Note that the MR results would remain similar if two taxa were considered a subcategory of the same phylum or another subcategory. Using the IVW method, the genus *LachnospiraceaeUCG004* exhibited the strongest protective association, showing a reduced likelihood of critical care admission (OR = 0.509, *P* = 8.84E-04). This association was consistently supported by multiple MR methods, including BWMR (OR = 0.496, *P* = 1.35E-03), maximum likelihood (OR = 0.494, *P* = 8.48E-04), and MR-PRESSO (OR = 0.509, *P* = 8.66E-03). Interestingly, the *Lentisphaeria* class/*Victivallales* order demonstrated a severity-dependent protective effect, wherein the strength of protection increased with more severe sepsis phenotypes. Under the IVW model, the odds ratios were 0.859 for sepsis (*P* = 1.65E-03), 0.678 for 28-day mortality in sepsis (*P* = 9.81E-04), 0.673 for sepsis requiring critical care (*P* = 4.32E-03), and 0.539 for 28-day mortality among critically ill septic patients (*P* = 2.63E-02). Importantly, the associations between *Lentisphaeria*/*Victivallales* and both sepsis and 28-day mortality remained statistically significant after multiple testing correction. To assess the robustness of these findings, we conducted extensive sensitivity analyses. As summarized in Supplementary Table S3, all 9 MR methods yielded consistent effect directions. Cochran’s Q test indicated no evidence of heterogeneity. The MR-Egger intercept and the MR-PRESSO global test did not suggest the presence of horizontal pleiotropy. Finally, the Steiger directionality test excluded the possibility of reverse causation.

#### 3.1.3 Causal effects of blood metabolites on sepsis

**FIGURE 2:**
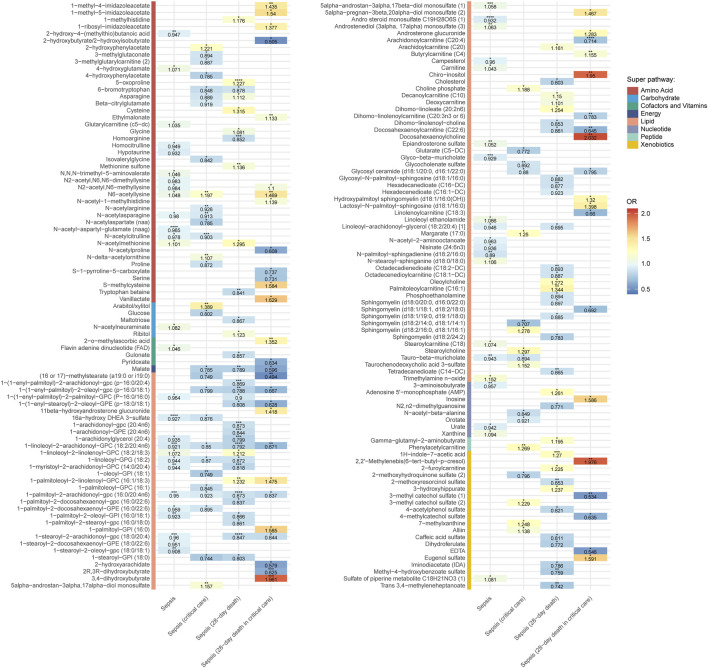
Heatmap showing the Mendelian randomization results between blood metabolites and sepsis and sepsis-related outcomes using inverse variance weighted method. OR, odds ratio. Red represents risk factor; blue represents protective factor. These metabolites could be classified into eight categories including lipid, amino acid, xenobiotics, nucleotide, cofactor and vitamins, carbohydrate, peptide, and energy. Each color block corresponds to a metabolic category. ^*^ *P* < 0.05, ^**^ *P* < 0.01, ^***^ *P* < 0.001, ^****^ *P* < 0.0001.

We estimated the causal effects of 850 blood metabolites on sepsis and sepsis-related outcomes and identified 206 significant causal associations, corresponding to 169 unique metabolites (Figure 2; Supplementary Table S4). A summary of these metabolites is provided in Supplementary Table S5. For consistency and ease of interpretation, common metabolite names were used throughout the study. Among the 169 metabolites, more than half (n = 86) belonged to lipid metabolism pathways, followed by 43 metabolites involved in amino acid metabolism, 20 in xenobiotic metabolism, 8 in nucleotide metabolism, 5 in carbohydrate metabolism, 4 in cofactors and vitamins metabolism, 2 in peptide metabolism, and 1 in energy metabolism. Seven of the causal associations remained statistically significant after multiple testing correction. These included: causal associations between N-acetylaspartylglutamic acid and sepsis (OR = 0.965, *P* = 2.30E-10); PC(18:0/20:4 (5Z,8Z,11Z,14Z)) and 28-day death in sepsis (OR = 0.847, *P* = 2.60E-08); pyroglutamic acid and 28-day death in sepsis (OR = 1.227, *P* = 1.49E-05); PC(18:2 (9Z,12Z)/20:4 (5Z,8Z,11Z,14Z)) and 28-day death in sepsis (OR = 0.792, *P* = 2.33E-05); PC(16:0/20:4 (5Z,8Z,11Z,14Z)) and 28-day death in sepsis (OR = 0.873, *P* = 4.53E-05); arachidonoylcarnitine and 28-day death in sepsis requiring critical care admission (OR = 0.714, *P* = 5.36E-05); 16alpha-Hydroxy DHEA 3-sulfate and sepsis (OR = 0.927, *P* = 5.71E-05). It’s worth noting that PC(18:2 (9Z,12Z)/20:4 (5Z,8Z,11Z,14Z)) has a severity-dependent protective effect on sepsis, with the OR for sepsis was 0.921 (*P* = 2.45E-04); for 28-day death in sepsis was 0.792 (*P* = 2.33E-05), for sepsis requiring critical care admission was 0.850 (*P* = 1.18E-02), and for 28-day death in sepsis requiring critical care admission was 0.671 (*P* = 2.06E-03). A similar effect was also produced by PC(16:0/20:4 (5Z,8Z,11Z,14Z)). Sensitivity analyses supported the robustness of these findings (Supplementary Table S4). All MR methods showed consistent effect directions. Cochran’s Q test revealed no evidence of heterogeneity, and both the MR-Egger intercept and MR-PRESSO global test indicated the absence of horizontal pleiotropy. The Steiger directionality test confirmed that the causal direction was correctly inferred.

**FIGURE 3:**
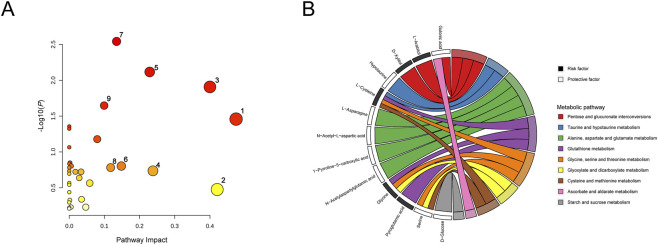
Identification of potential metabolic pathways. **(A)** Metabolic pathway enrichment analysis 1. Glycine, serine and threonine metabolism; 2. Starch and sucrose metabolism; 3. Taurine and hypotaurine metabolism; 4. Ascorbate and aldarate metabolism; 5. Pentose and glucuronate interconversions; 6. Glyoxylate and dicarboxylate metabolism; 7. Alanine, aspartate and glutamate metabolism; 8. Cysteine and methionine metabolism; 9. Glutathione metabolism. **(B)** Chord diagram showing the mapping relationships between the nine pathways and corresponding metabolites.

To further elucidate metabolic mechanisms underlying sepsis, the 169 metabolites were analyzed using the MetaboAnalyst platform for pathway enrichment. As shown in Figure 3A, nine metabolic pathways were significantly enriched (impact value >0.1). Among these, glycine, serine and threonine metabolism, starch and sucrose metabolism, taurine and hypotaurine metabolism, ascorbate and aldarate metabolism, and pentose and glucuronate interconversions were particularly important. A chord (string) diagram illustrated the mapping relationships between these pathways and their corresponding metabolites (Figure 3B).

#### 3.1.4 Mediation mendelian randomization analysis

**FIGURE 4:**
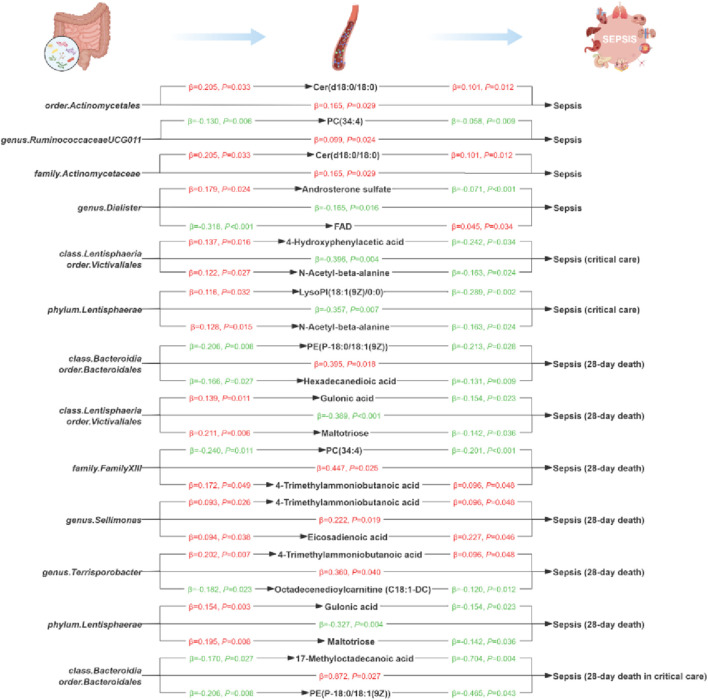
Mediation Mendelian randomization analyses of blood metabolites between gut microbiota and sepsis and sepsis-related outcomes. The diagram displays the mediation mode of “gut microbiota-blood metabolite-sepsis” in two-step Mendelian randomization. β indicates the causal effect estimate using inverse variance weighted method. Characters colored in red and green signify positive and negative associations respectively.

Based on the above findings, for each sepsis outcome we identified GM taxa that were causally associated with that outcome and, in parallel, blood metabolites that were also causally associated with the same outcome. These metabolite sets were therefore taken forward as candidate mediators for constructing the GM-metabolite-sepsis outcome network. Specifically, 10 GM taxa and 48 metabolites were associated with sepsis, 6 GM taxa and 46 metabolites with sepsis requiring critical care, 10 GM taxa and 65 metabolites with 28-day mortality in sepsis, and 9 GM taxa and 47 metabolites with 28-day mortality in critical-care sepsis (Supplementary Tables S3, S4). Accordingly, we further evaluated the causal relationships between specific GM taxa and metabolites for each of the four sepsis outcomes (Supplementary Table S6). A two-step MR analysis was subsequently conducted to investigate the mediating network through which GM taxa influence sepsis and its related outcomes via blood metabolites. The results revealed that the causal effects of 12 GM taxa on sepsis phenotypes may be partially mediated by 15 specific blood metabolites (Figure 4; Table 2). Of these metabolites, ten belonged to lipid metabolism pathways, two to cofactors and vitamins, one to amino acid metabolism, one to nucleotide metabolism, and one to carbohydrate metabolism, highlighting a predominant mediating role of lipid metabolites in GM-sepsis interactions. For example, the causal association between the *Bacteroidia* class/*Bacteroidales* order and 28-day mortality in critical-care sepsis was mediated by 17-methyloctadecanoic acid, with a mediation proportion of 13.7%. In addition, both the *Actinomycetaceae* family and *Actinomycetales* order exerted detrimental effects on sepsis by increasing Cer(d18:0/18:0) levels, with a mediation proportion of 12.5%. Interestingly, 4-trimethylammoniobutanoic acid appeared most frequently within the mediation network, linking several GM taxa to 28-day sepsis mortality with mediation proportions ranging from 3.70% to 5.40%. Taken together, the mediation analysis established a mechanistic bridge connecting the GM, circulating metabolites, and sepsis-related outcomes, providing genetic evidence for a GM-metabolite-sepsis axis.

**TABLE 2:** Mediation analyses of blood metabolites between gut microbiota and sepsis and sepsis-related outcomes.

Outcome	Exposure	Mediator	β_0_±SE	β_1_±SE	β_2_±SE	Indirect effect (β_1_*β_2_±SE)	Proportion mediated (β_1_*β_2_/β_0_)
Sepsis	family. Actinomycetaceae	Cer(d18:0/18:0)	0.165 ± 0.076	0.205 ± 0.096	0.101 ± 0.040	0.021 ± 0.013	0.125
	genus. Dialister	Androsterone sulfate	−0.165 ± 0.068	0.179 ± 0.079	−0.071 ± 0.018	−0.013 ± 0.007	0.077
	genus. Dialister	FAD	−0.165 ± 0.068	−0.318 ± 0.089	0.045 ± 0.021	−0.014 ± 0.008	0.086
	genus. RuminococcaceaeUCG011	PC(34:4)	0.099 ± 0.044	−0.130 ± 0.048	−0.058 ± 0.022	0.008 ± 0.004	0.077
	order. Actinomycetales	Cer(d18:0/18:0)	0.165 ± 0.076	0.205 ± 0.096	0.101 ± 0.040	0.021 ± 0.013	0.125
Sepsis (critical care)	class. Lentisphaeria/order. Victivallales	4-Hydroxyphenylacetic acid	−0.396 ± 0.139	0.137 ± 0.057	−0.242 ± 0.115	−0.033 ± 0.022	0.084
	class. Lentisphaeria/order.Victivallales	N-Acetyl-beta-alanine	−0.396 ± 0.139	0.122 ± 0.055	−0.163 ± 0.073	−0.020 ± 0.013	0.050
	phylum. Lentisphaerae	LysoPI(18:1 (9Z)/0:0)	−0.357 ± 0.132	0.116 ± 0.054	−0.289 ± 0.091	−0.034 ± 0.020	0.094
	phylum. Lentisphaerae	N-Acetyl-beta-alanine	−0.357 ± 0.132	0.128 ± 0.052	−0.163 ± 0.073	−0.021 ± 0.013	0.058
Sepsis (28-day death)	class. Bacteroidia/order. Bacteroidales	PE (P-18:0/18:1 (9Z))	0.395 ± 0.168	−0.206 ± 0.078	−0.213 ± 0.097	0.044 ± 0.027	0.111
	class. Bacteroidia/order. Bacteroidales	Hexadecanedioic acid	0.395 ± 0.168	−0.166 ± 0.075	−0.131 ± 0.050	0.022 ± 0.013	0.055
	class. Lentisphaeria/order. Victivallales	Gulonic acid	−0.389 ± 0.118	0.139 ± 0.054	−0.154 ± 0.068	−0.021 ± 0.013	0.055
	class. Lentisphaeria/order. Victivallales	Maltotriose	−0.389 ± 0.118	0.211 ± 0.077	−0.142 ± 0.068	−0.030 ± 0.019	0.077
	family. FamilyXIII	PC(34:4)	0.447 ± 0.199	−0.240 ± 0.094	−0.201 ± 0.054	0.048 ± 0.023	0.108
	family. FamilyXIII	4-Trimethylammoniobutanoic acid	0.447 ± 0.199	0.172 ± 0.087	0.096 ± 0.049	0.017 ± 0.013	0.037
	genus. Sellimonas	Eicosadienoic acid	0.222 ± 0.094	0.094 ± 0.045	0.227 ± 0.114	0.021 ± 0.016	0.096
	genus. Sellimonas	4-Trimethylammoniobutanoic acid	0.222 ± 0.094	0.093 ± 0.042	0.096 ± 0.049	0.009 ± 0.006	0.040
	genus. Terrisporobacter	Octadecenedioylcarnitine (C18:1-DC)	0.360 ± 0.176	−0.182 ± 0.080	−0.120 ± 0.048	0.022 ± 0.014	0.060
	genus. Terrisporobacter	4-Trimethylammoniobutanoic acid	0.360 ± 0.176	0.202 ± 0.075	0.096 ± 0.049	0.019 ± 0.013	0.054
	phylum. Lentisphaerae	Gulonic acid	−0.327 ± 0.112	0.154 ± 0.051	−0.154 ± 0.068	−0.024 ± 0.014	0.073
	phylum. Lentisphaerae	Maltotriose	−0.327 ± 0.112	0.195 ± 0.073	−0.142 ± 0.068	−0.028 ± 0.018	0.085
Sepsis (28-day death in critical care)	class. Bacteroidia/order. Bacteroidales	17-Methyloctadecanoic acid	0.872 ± 0.394	−0.170 ± 0.077	−0.704 ± 0.241	0.120 ± 0.070	0.137
	class. Bacteroidia/order. Bacteroidales	PE (P-18:0/18:1 (9Z))	0.872 ± 0.394	−0.206 ± 0.078	−0.465 ± 0.230	0.096 ± 0.062	0.110

SE, standard error. β_0_ is the total effect of gut microbiota on sepsis and sepsis-related outcomes; β_1_ is the causal effect of gut microbiota on blood metabolites; β_2_ is the causal effect of blood metabolites on sepsis and sepsis-related outcomes. Proportion mediated is calculated as the “indirect effect/total effect”.

### 3.2 Network pharmacology analysis

#### 3.2.1 Construction of a sepsis-target-metabolite network

**FIGURE 5:**
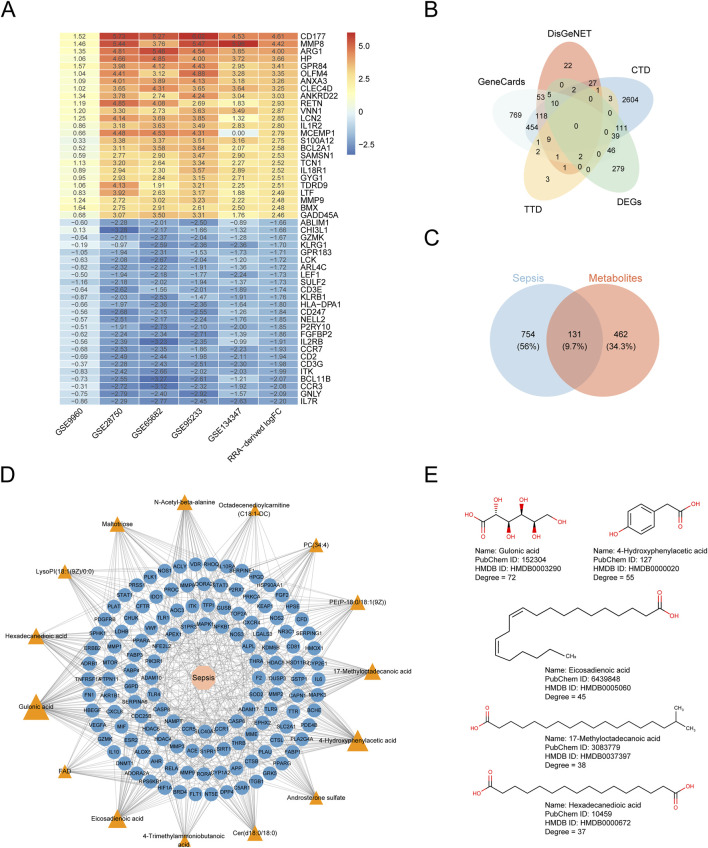
Construction of the sepsis-target-metabolite network. **(A)** Heatmap displaying the top 25 upregulated and top 25 downregulated differentially expressed genes identified by robust rank aggregation analysis. Blue represents downregulation and red represents upregulation. The numbers shown on the heatmap denote the log fold-change (logFC) values of each gene calculated in the corresponding dataset. **(B)** Venn diagram of sepsis-related targets from four disease databases and differentially expressed genes. **(C)** Venn diagram showing the overlaps between sepsis-related targets and mediator metabolites-related targets. **(D)** The sepsis-target-metabolite interaction network. The triangle represents the mediator metabolite. The blue circle represents the overlapping target. **(E)** The two-dimensional chemical structures and information of five critical metabolites.

A total of 273, 155, 156, and 135 potential targets of the 15 mediator metabolites were predicted using the Super-PRED, PharmMapper, STP, and TargetNet databases, respectively. After merging the results and removing duplicates, 593 unique metabolite-related targets were retained. For sepsis, 251, 1,509, 23, and 3,379 potential therapeutic targets were identified from the DisGeNET, GeneCards, TTD, and CTD databases, respectively. In addition, 494 DEGs were identified based on RRA-derived *P* < 0.05 and |logFC| > 1. The logFC values of the top 25 upregulated and top 25 downregulated genes across datasets are shown in Figure 5A. Sepsis-related targets that appeared in at least two of the above databases were selected and intersected with the 593 metabolite-related targets (Figure 5B) (Ji et al., 2023). This process yielded 131 shared targets (Figure 5C). A sepsis-target-metabolite interaction network was subsequently constructed using Cytoscape (Figure 5D). In this network, the Degree value reflected the number of sepsis-associated targets modulated by each metabolite. Strikingly, gulonic acid (GA), 4-hydroxyphenylacetic acid (4-HPA), eicosadienoic acid, 17-methyloctadecanoic acid, and hexadecanedioic acid ranked among the top five metabolites with the highest Degree values, indicating that these metabolites have the strongest potential regulatory influence on sepsis progression. The chemical structures of these five metabolites are presented in Figure 5E.

#### 3.2.2 Construction and analysis of a PPI network

**FIGURE 6:**
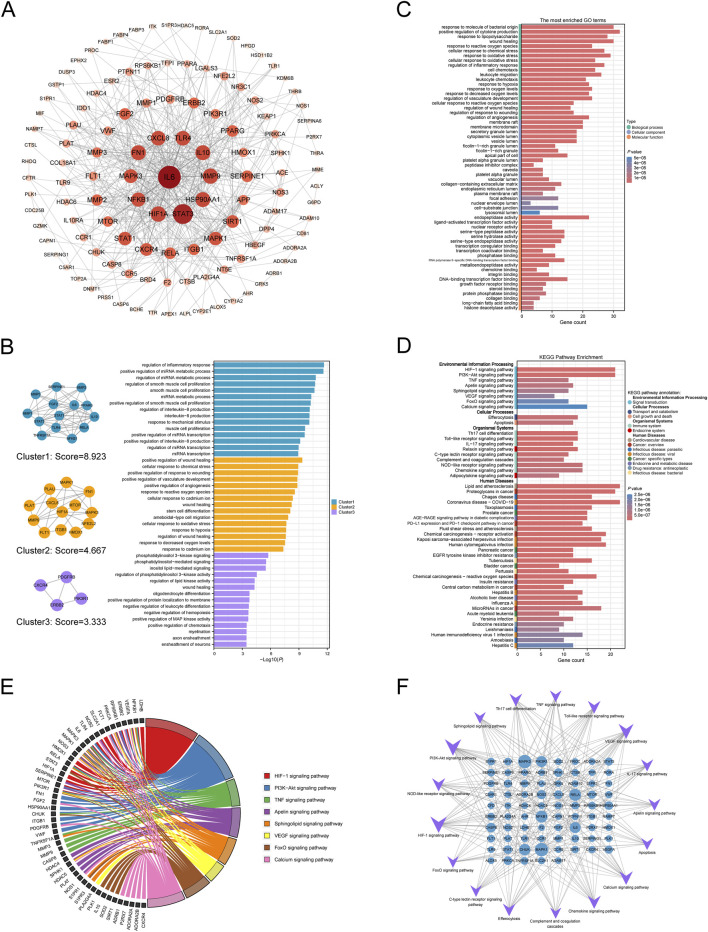
Protein-protein interaction network construction and functional enrichment analysis of the overlapping targets. **(A)** Protein-protein interaction network. **(B)** The three protein clusters obtained by MCODE plug-in and GO-biological process enrichment analysis for each protein cluster. **(C)** GO enrichment analysis of the overlapping targets. The top 20 terms of biological process, molecular function and cellular component are shown. **(D)** KEGG enrichment analysis of the overlapping targets. The top 50 results are shown. **(E)** Chord diagram of the eight pathways involved in the signal transduction section. **(F)** Target-pathway network.

The 131 shared targets were imported into the STRING database to construct a PPI network, which consisted of 121 nodes and 515 edges (Figure 6A). Notably, the network was dominated by an inflammation-centered module, consistent with sepsis being driven by a dysregulated host response to infection (Hotchkiss et al., 2016). Using the cytoHubba plug-in, the importance scores of each target were calculated using four topological algorithms (Degree, MCC, Closeness, and Betweenness), and the top 30 targets ranked by Degree are presented in Table 3. Among these, IL6, STAT3, CXCL8, MMP9, and MMP1 were identified as core targets based on the MCC algorithm. Subsequently, protein clustering was performed using the MCODE plug-in, yielding three distinct protein clusters (Figure 6B). GO-BP enrichment analysis was then conducted for each cluster. Cluster 1, centered on the seed protein STAT1, was primarily enriched in pathways related to the regulation of inflammatory responses and the positive regulation of miRNA metabolic processes, suggesting that interferon/STAT1-linked inflammatory programs and post-transcriptional regulation may jointly shape the septic immune response (Formosa et al., 2022). Cluster 2, seeded by PLAU, was enriched in pathways associated with the positive regulation of wound healing and cellular responses to chemical stress, pointing to the plasminogen/urokinase system and tissue repair pathways that are frequently perturbed during sepsis and related coagulopathy/vascular injury (Napolitano et al., 2023). Cluster 3 was mainly involved in phosphatidylinositol 3-kinase signaling and phosphatidylinositol-mediated signaling, supporting a role for PI3K-related signaling in coordinating inflammatory injury with endothelial/organ dysfunction during sepsis (Chen K. et al., 2024).

**TABLE 3:** Intersection targets with the top 30°.

Targets	Degree	MCC	Betweenness	Closeness
IL6	48	194274	2371.88221	80.08333
STAT3	40	174386	1776.29022	76.58333
CXCL8	26	152644	591.94183	66.41667
MMP9	28	143604	265.60903	65.75
MMP1	11	143334	608.72625	67.91667
IL10	27	116174	843.20575	67.66667
FN1	27	100800	5.1248	55.58333
FGF2	18	100338	117.13169	61.91667
NFKB1	25	77360	480.93001	67.16667
STAT1	23	61878	321.68596	65.75
MMP2	17	56490	172.44209	61.16667
MMP3	14	51133	92.18796	58.08333
HIF1A	28	49886	1087.18124	68.58333
TLR4	26	40135	599.62743	67.58333
RELA	18	36375	414.81898	61.5
PPARG	18	32683	412.84592	62.08333
SERPINE1	18	21336	375.00296	59.36667
MAPK1	23	12570	879.23436	66.5
CXCR4	21	10295	858.37466	64.25
MTOR	15	7752	72.52049	59.83333
MAPK3	24	6138	889.53662	66.58333
ERBB2	16	2480	148.59255	60.25
CCR5	11	2403	337.10077	54.91667
SIRT1	21	2388	645.39779	63.16667
HSP90AA1	27	2384	1895.69584	68.91667
PDGFRB	11	1848	17.63641	55.83333
HMOX1	12	1118	98.33367	56.58333
ITGB1	17	935	481.21493	61.58333
PIK3R1	13	303	487.97233	57.33333
APP	14	134	1009.61168	58.86667

#### 3.2.3 GO and KEGG enrichment analyses for overlapping targets

GO enrichment of the overlapping targets further supported an infection-triggered inflammatory mechanism. Specifically, the top biological processes, response to molecule of bacterial origin, response to lipopolysaccharide, and positive regulation of cytokine production (Figure 6C), are core components of the host response to bacterial infection in sepsis (Hotchkiss et al., 2016). The enriched cellular components (membrane raft/microdomain) suggest that many targets participate in receptor-proximal innate immune signaling platforms, as lipid rafts are important for organizing LPS-TLR4 signaling and downstream inflammatory cascades (Płóciennikowska et al., 2015). The dominant molecular functions (endopeptidase activity and nuclear receptor activity) indicate two additional layers of regulation: proteolytic remodeling (e.g., MMP-related activity) that can contribute to tissue damage and vascular leakage, and metabolite-sensing transcriptional regulation via nuclear receptors that link systemic metabolism to inflammation. KEGG enrichment (Figure 6D) showed that these targets converge on signaling pathways repeatedly implicated in sepsis pathophysiology, including TNF and PI3K-Akt signaling (inflammation and cell survival), HIF-1 signaling (hypoxia/inflammatory adaptation), VEGF signaling (vascular permeability), and apoptosis/efferocytosis-related processes (cell death and inflammatory resolution). The interaction diagram of the eight selected signaling pathways (Figure 6E) and the target–pathway network (Figure 6F; Table 4) together indicate that these pathways are coupled through shared hub targets, suggesting that mediator metabolites may influence sepsis not through a single linear route but by coordinating multiple interconnected inflammatory, hypoxia/vascular, and cell-fate programs.

**TABLE 4:** The targets of 17 signaling pathways related to sepsis.

ID	Description	Count	P value	geneID
hsa04066	HIF-1 signaling pathway	21	1.11E-18	LDHB, NFKB1, VEGFA, ERBB2, RPS6KB1, PRKCA, FLT1, SLC2A1, NOS2, TLR4, IL6, MAPK3, MAPK1, NOS3, HMOX1, RELA, STAT3, HIF1A, SERPINE1, MTOR, PIK3R1
hsa04151	PI3K-Akt signaling pathway	21	2.46E-08	NFKB1, VEGFA, ERBB2, FN1, FGF2, RPS6KB1, PRKCA, HSP90AA1, CHUK, FLT1, ITGB1, PDGFRB, TLR4, IL6, MAPK3, MAPK1, NOS3, RELA, MTOR, VWF, PIK3R1
hsa04020	Calcium signaling pathway	15	2.51E-06	ADRB1, VEGFA, ERBB2, FGF2, PRKCA, FLT1, SPHK1, PDGFRB, P2RX7, NOS2, NOS3, ADORA2A, ADORA2B, NOS1, CXCR4
hsa04621	NOD-like receptor signaling pathway	14	3.21E-07	NFKB1, HSP90AA1, CHUK, CTSB, P2RX7, NAMPT, TLR4, IL6, MAPK3, MAPK1, CXCL8, RELA, STAT1, CASP8
hsa04062	Chemokine signaling pathway	14	4.73E-07	NFKB1, CHUK, GRK5, CCR1, CCR5, ITK, MAPK3, MAPK1, CXCL8, RELA, STAT1, STAT3, CXCR4, PIK3R1
hsa04620	Toll-like receptor signaling pathway	13	3.14E-09	NFKB1, CHUK, TLR1, TLR4, TLR9, IL6, MAPK3, MAPK1, CXCL8, RELA, STAT1, CASP8, PIK3R1
hsa04659	Th17 cell differentiation	13	3.14E-09	RORA, NFKB1, HSP90AA1, CHUK, AHR, IL6, MAPK3, MAPK1, RELA, STAT1, STAT3, HIF1A, MTOR
hsa04148	Efferocytosis	13	2.68E-07	PPARG, SLC2A1, SPHK1, ADAM17, ALOX5, PTPN11, S1PR1, ADAM10, MAPK3, MAPK1, IL10, HIF1A, SIRT1
hsa04657	IL-17 signaling pathway	12	6.64E-09	NFKB1, MMP3, HSP90AA1, CHUK, MMP9, IL6, MAPK3, MAPK1, CXCL8, RELA, CASP8, MMP1
hsa04210	Apoptosis	12	3.95E-07	CASP6, NFKB1, TNFRSF1A, CTSL, CHUK, CAPN1, CTSB, MAPK3, MAPK1, RELA, CASP8, PIK3R1
hsa04371	Apelin signaling pathway	12	5.42E-07	HDAC4, RPS6KB1, SPHK1, HDAC5, NOS2, MAPK3, MAPK1, NOS3, PLAT, SERPINE1, NOS1, MTOR
hsa04625	C-type lectin receptor signaling pathway	11	2.11E-07	NFKB1, CHUK, PTPN11, IL6, MAPK3, MAPK1, IL10, RELA, STAT1, CASP8, PIK3R1
hsa04668	TNF signaling pathway	11	5.40E-07	NFKB1, TNFRSF1A, MMP3, CHUK, MMP9, IL6, MAPK3, MAPK1, RELA, CASP8, PIK3R1
hsa04071	Sphingolipid signaling pathway	11	9.85E-07	NFKB1, TNFRSF1A, PRKCA, SPHK1, S1PR1, S1PR3, MAPK3, MAPK1, NOS3, RELA, PIK3R1
hsa04068	FoxO signaling pathway	11	2.17E-06	CHUK, PLK1, S1PR1, IL6, MAPK3, MAPK1, IL10, SOD2, STAT3, SIRT1, PIK3R1
hsa04610	Complement and coagulation cascades	10	3.15E-07	PLAU, SERPING1, PROC, C5AR1, TFPI, CFD, PLAT, SERPINE1, VWF, F2
hsa04370	VEGF signaling pathway	8	1.54E-06	PLA2G4A, VEGFA, PRKCA, SPHK1, MAPK3, MAPK1, NOS3, PIK3R1

#### 3.2.4 Construction of a GMTS network

**FIGURE 7:**
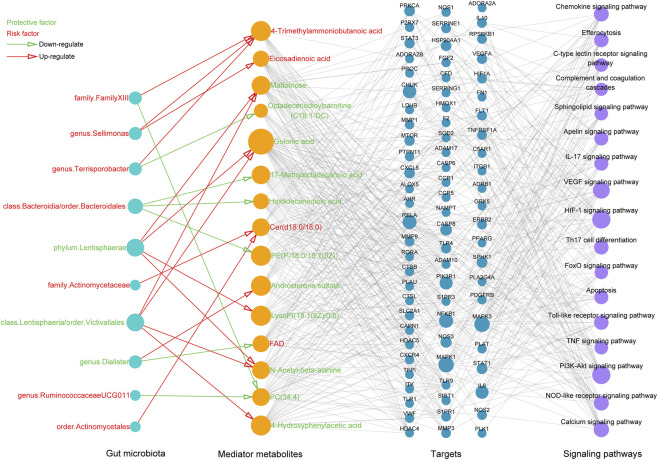
Network visualization highlighting the detailed interactions among sepsis, gut microbiota, mediator metabolites, targets, and signaling pathways. Bright blue circle represents gut microbiota; yellow circle represents metabolite; blue circle represents target; purple circle represents signaling pathway involved. Characters colored in red and green signify risk and protective factors of sepsis, respectively. Open arrows colored in red and green signify positive and negative regulation respectively.

By integrating the GM-metabolite-sepsis mediation network, the sepsis-target-metabolite interaction network, and the target-pathway network in Cytoscape, we constructed a comprehensive GM- Metabolite-Target-Signaling (GMTS) network. This network depicts the complex relationships among sepsis, 12 GM taxa (bright blue nodes), 15 mediator metabolites (yellow nodes), 79 shared targets (blue nodes), and 17 enriched signaling pathways (purple nodes) (Figure 7). Based on node Degree values, the *Lentisphaerae* phylum and *Lentisphaeria* class/*Victivallales* order emerged as key microbial taxa capable of influencing sepsis by simultaneously regulating four mediator metabolites. As expected from earlier analyses, GA and 4-HPA displayed the highest Degrees among metabolites, supporting their role as major protective factors. In network biology, targets involved in a greater number of pathways typically play more central roles in disease progression. Consistent with this principle, MAPK1 and MAPK3 were found to participate in nearly all identified signaling pathways, underscoring their importance as key regulatory hubs in sepsis. Taken together, this integrative GMTS network highlights that the gut microbiota, blood metabolites, molecular targets, and signaling pathways act in concert to influence sepsis progression from a systems-level perspective.

### 3.3 Molecular docking assay

**FIGURE 8:**
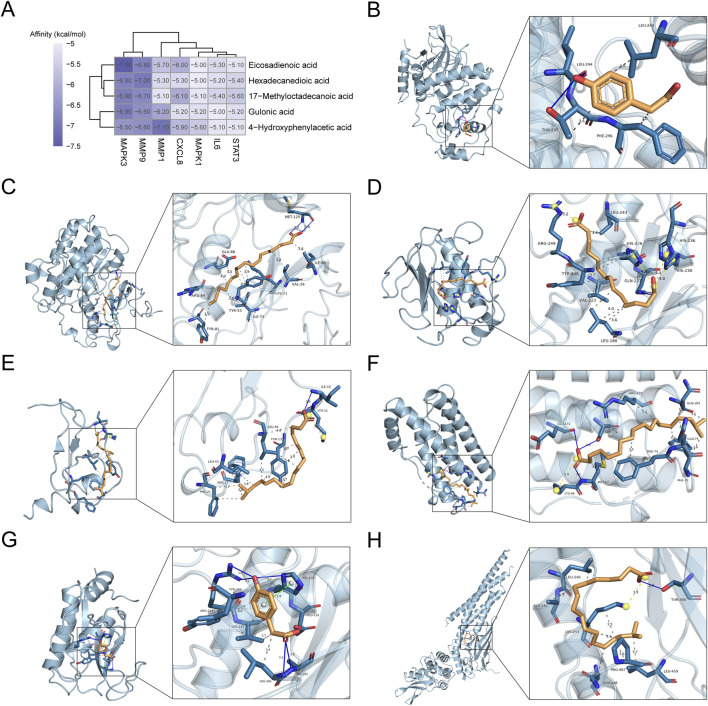
The results of molecular docking. **(A)** Heatmap showing the molecular docking results. The three-dimensional views of the optimal docking, including **(B)** 4-HPA-MAPK1, **(C)** eicosadienoic acid-MAPK3, **(D)** hexadecanedioic acid-MMP9, **(E)** 17-methyloctadecanoic acid-CXCL8, **(F)** 17-methyloctadecanoic acid-IL6, **(G)** 4-HPA-MMP1, and **(H)** 17-methyloctadecanoic acid-STAT3.

According to the above analyses, the five critical mediator metabolites (GA, 4-HPA, eicosadienoic acid, 17-methyloctadecanoic acid, and hexadecanedioic acid) were selected for molecular docking with seven core targets including IL6, CXCL8, MMP9, MMP1, STAT3, MAPK1, and MAPK3. As previously stated, affinity < −4.25 kcal/mol implies the presence of a binding activity; < −5.0 kcal/mol indicates a good binding activity; < −7.0 kcal/mol indicates a strong docking activity (Hsin et al., 2013). As shown in Figure 8A, all target-ligand pairs exhibited good binding activities, which demonstrated the reliability of network pharmacology. Figures 8B–H displays the optimal docking results of each target, including 4-HPA-MAPK1 (−5.6), eicosadienoic acid-MAPK3 (−7.5), hexadecanedioic acid-MMP9 (−7.0), 17-methyloctadecanoic acid-CXCL8 (−6.1), 17-methyloctadecanoic acid-IL6 (−5.4), 4-HPA-MMP1 (−7.1), and 17-methyloctadecanoic acid-STAT3 (−5.6).

### 3.4 GA and 4-HPA intervention in LPS-induced mice

#### 3.4.1 Effects of GA and 4-HPA on survival rate and severity score in LPS-induced mice

**FIGURE 9:**
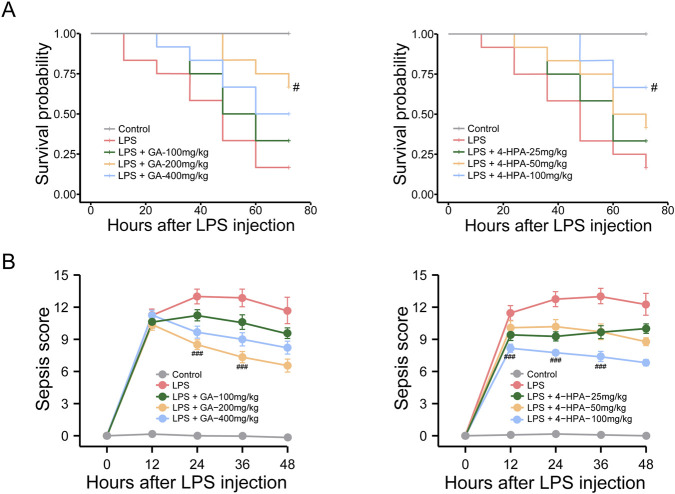
Effects of GA and 4-HPA on survival rate and murine sepsis score (MMS) in LPS-induced mice. **(A)** The Kaplan-Meier survival curves assessed for up to 72 h. Each line represents the survival of mice in a group; 12 mice were in each group. **(B)** MMS assessed for up to 48 h. ^#^ *P* < 0.05, ^##^ *P* < 0.01, ^###^ *P* < 0.001 *versus* LPS group.

Integrating the above findings, GA and 4-HPA were identified as the most promising GM-derived metabolites for potential sepsis therapy. Accordingly, we conducted pharmacodynamic experiments to determine whether exogenous administration of GA or 4-HPA could ameliorate LPS-induced injury in mice. First, various doses of GA (100, 200, 400 mg/kg) and 4-HPA (25, 50, 100 mg/kg) were administered to assess their effects on survival in an LPS-induced sepsis model. As shown in Figure 9A, LPS exposure resulted in high mortality (∼80%), whereas treatment with either GA or 4-HPA markedly improved survival. Moreover, both metabolites significantly reduced the MSS compared with the LPS group, indicating a clear attenuation of sepsis severity (Figure 9B). Notably, 200 mg/kg GA and 100 mg/kg 4-HPA produced the most pronounced protective effects and were therefore selected for subsequent experiments.

#### 3.4.2 Effects of GA and 4-HPA on LPS-induced multiple organ damage

**FIGURE 10:**
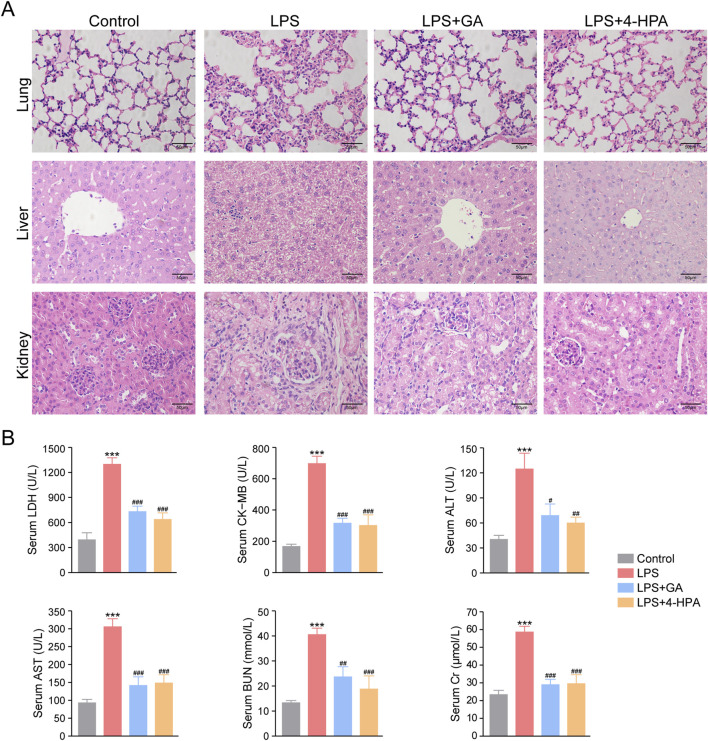
Effects of GA and 4-HPA on multiple organ injury in LPS-stimulated mice. **(A)** Hematoxylin and eosin staining for lung, liver, and kidney tissues. **(B)** Blood biochemical indexes. ^*^ *P* < 0.05, ^**^ *P* < 0.01, ^***^ *P* < 0.001 *versus* Control group; ^#^ *P* < 0.05, ^##^ *P* < 0.01, ^###^ *P* < 0.001 *versus* LPS group.

Multiple organ injury is a defining feature of sepsis; therefore, histopathological analyses were performed on major organs from LPS-induced septic mice. As shown in Figure 10A, the lung, liver, and kidney tissues of LPS-treated mice exhibited pronounced pathological abnormalities, including hemorrhage, inflammatory cell infiltration, cell death, vacuolar degeneration, and edema. Notably, treatment with either GA or 4-HPA markedly alleviated these histological alterations. Consistent with the histological findings, both GA and 4-HPA significantly reduced the LPS-induced increases in serum CK-MB, LDH, ALT, AST, BUN, and Cr levels (Figure 10B). Collectively, these results demonstrate that GA and 4-HPA effectively mitigate multi-organ injury in septic mice.

#### 3.4.3 Effects of GA and 4-HPA on systemic inflammation in LPS-induced mice

**FIGURE 11:**
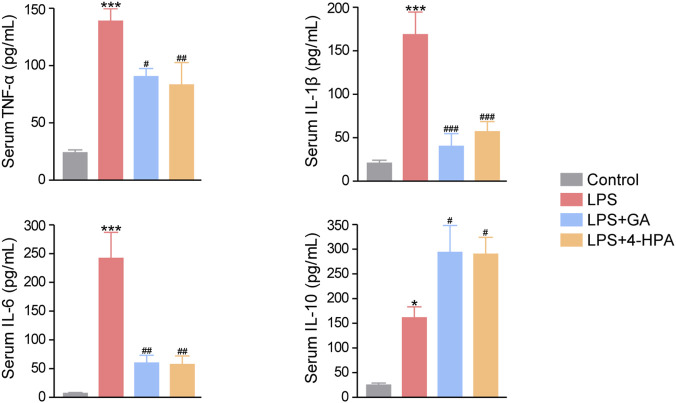
Effects of GA and 4-HPA on inflammation response in LPS-induced mice. ^*^ *P* < 0.05, ^**^ *P* < 0.01, ^***^ *P* < 0.001 *versus* Control group; ^#^ *P* < 0.05, ^##^ *P* < 0.01, ^###^ *P* < 0.001 *versus* LPS group.

We next examined the effects of GA and 4-HPA on systemic inflammation in septic mice. ELISA results showed that serum levels of the proinflammatory cytokines TNF-α, IL-6, and IL-1β were markedly elevated in the LPS group compared with the control group. Conversely, the anti-inflammatory cytokine IL-10 was also increased in septic mice, reflecting a compensatory regulatory response. Treatment with GA or 4-HPA significantly reduced the levels of TNF-α, IL-6, and IL-1β, while further enhancing the production of IL-10 (Figure 11). These findings indicate that both metabolites effectively suppress systemic inflammation in septic mice.

#### 3.4.4 Effects of GA and 4-HPA on the expression of core targets

**FIGURE 12:**
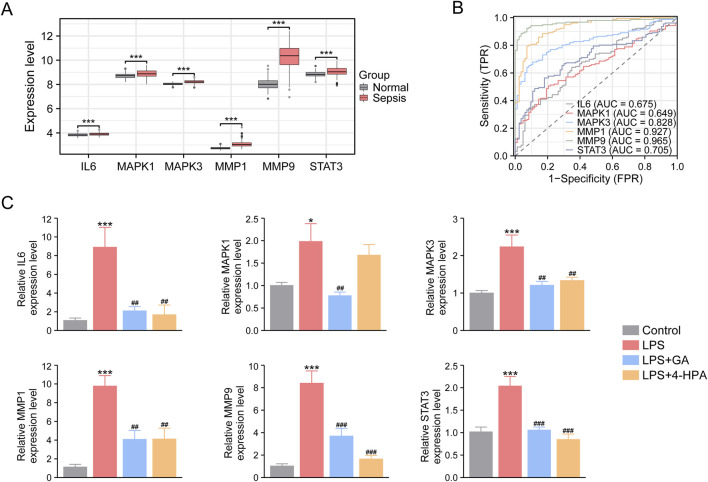
Effects of GA and 4-HPA on the expression of core targets. **(A)** Differential expression analysis of the core targets using GSE134347 dataset. **(B)** ROC curves showing the predicted value of core targets. **(C)** The mRNA expression of core targets in liver tissues of mice. ^*^ *P* < 0.05, ^**^ *P* < 0.01, ^***^ *P* < 0.001 *versus* Control group; ^#^ *P* < 0.05, ^##^ *P* < 0.01, ^###^ *P* < 0.001 *versus* LPS group.

Based on the GSE134347 dataset, the expression levels of the six core targets, including IL6, MMP9, MMP1, STAT3, MAPK1, and MAPK3, were markedly elevated in septic patients compared with healthy individuals (Figure 12A). Receiver operating characteristic (ROC) curve analysis further demonstrated the strong predictive performance of these core genes for identifying sepsis (Figure 12B). To experimentally validate these findings, we measured the expression of the six genes using RT-qPCR. As shown in Figure 12C, all six targets were significantly upregulated in the septic group relative to the control group, whereas treatment with GA or 4-HPA effectively reversed these elevations. These results suggest that GA and 4-HPA may exert protective effects in sepsis by modulating the expression of these core regulatory targets.

## 4 Discussion

To our knowledge, no prior studies have reported that blood metabolites mediate the causal pathway between the GM and sepsis. In this large-scale MR analysis, we identified 23 GM taxa with potential causal influences on sepsis onset, progression, and mortality. More importantly, 15 specific blood metabolites were found to mediate the causal effects of 12 GM taxa on distinct sepsis outcomes through two-step MR analyses. Among these taxa, the *LachnospiraceaeUCG004* genus exhibited a negative causal association with sepsis requiring critical care. Previous studies have shown that *Lachnospiraceae* abundance decreases in the small intestine of septic mice (Peng et al., 2022) and that this family plays an important role in modulating intestinal inflammation (Sun et al., 2021; Zhang H. et al., 2021), likely through the production of short-chain fatty acids (SCFAs) (Wong et al., 2014). Furthermore, recent work revealed negative correlations between *Lachnospiraceae* abundance and organ damage, morbidity, and mortality in septic mice (Yu et al., 2021; Gai et al., 2021), consistent with our findings. The *Victivallales* order/*Lentisphaeria* class, belonging to the *Lentisphaerae* phylum, demonstrated a severity-dependent protective effect against sepsis in our study. This is biologically plausible, as *Lentisphaerae* have been implicated in maintaining gut integrity through contributions to intestinal mucus barrier formation (Shang et al., 2024). In contrast, our results reaffirmed the pro-inflammatory properties of *Gammaproteobacteria* and *Bacteroidales* (Arimatsu et al., 2014; Mukhopadhya et al., 2012; Amar et al., 2011), both of which increase sepsis susceptibility. The relationship between *Ruminococcaceae* and sepsis, however, remains controversial, with animal and clinical studies reporting inconsistent results (Muratsu et al., 2022; Stoma et al., 2021; Zhang et al., 2023). Likewise, our analysis revealed opposing effects of genera *RuminococcaceaeUCG011* and *Ruminiclostridium6*, suggesting that finer taxonomic resolution is required to accurately capture the contributions of *Ruminococcaceae* subgroups to sepsis risk. Finally, *Coprococcus*, an important SCFA-producing genus (Azcarate-Peril et al., 2021) and a recognized microbial biomarker of gut health (Yang et al., 2023; Shen et al., 2018), was negatively associated with sepsis risk in prior studies (Szabó et al., 2022; Yu et al., 2022), in agreement with our MR findings.

Regarding the mechanisms through which the GM influences sepsis, our study provides genetic evidence that 15 specific blood metabolites mediate the causal effects of 12 GM taxa on diverse sepsis outcomes. Notably, these mediators were predominantly lipid metabolites, underscoring the central role of lipid metabolism in the GM-sepsis axis. Lipids are well known to participate in inflammation and infection-induced organ injury (Bai and Guo, 2017), and significant alterations in circulating lipids have been consistently observed in septic patients and animal models (Ghosh and Nishtala, 2017; Park et al., 2014; Rival et al., 2013). However, only limited studies have directly linked specific GM taxa to their corresponding circulating metabolites. To elucidate the molecular underpinnings of these mediators, we constructed a target-metabolite interaction network containing 131 targets and 15 metabolites, from which several key metabolites with broad regulatory potential were identified, GA, 4-HPA, eicosadienoic acid, 17-methyloctadecanoic acid, and hexadecanedioic acid. MR analyses further showed that GA, 4-HPA, 17-methyloctadecanoic acid, and hexadecanedioic acid exerted protective effects on sepsis, whereas eicosadienoic acid had harmful effects. These metabolites mediated the causal influences of *Lentisphaeria* class/*Victivallales* order, *Bacteroidia* class/*Bacteroidales* order, *Sellimonas* genus, and the *Lentisphaerae* phylum. GA, a metabolite in the ascorbate/aldarate pathway, is produced via the reduction of glucuronic acid during inositol catabolism (Barski et al., 2005), and has recently been reported as a biomarker of kidney function (Denburg et al., 2021). GA is also a major component of Amaranthus viridis (Kumari et al., 2018) and *Centella asiatica* (Kumari et al., 2016), where it exhibits anti-inflammatory, antioxidant, and anti-hyperlipidemic activities. 4-HPA, a major colonic microbiota-derived metabolite generated through microbial biotransformation of plant compounds (An et al., 2024), circulates in human blood (Jenner et al., 2005) and possesses multiple pharmacological activities, including anxiolytic (Vissiennon et al., 2012), antiplatelet (Kim et al., 1998), and organ-protective effects. Its protective influence has been documented in acetaminophen-induced hepatic injury (Zhao et al., 2018), seawater-induced lung injury (Liu et al., 2014), and more recently, sepsis-induced acute kidney injury (An et al., 2024), consistent with our findings. 17-Methyloctadecanoic acid (isononadecanoic acid) is a monomethyl branched-chain fatty acid (mmBCFA) (Kniazeva et al., 2004). Unlike cytotoxic and pro-inflammatory saturated fatty acids (SFAs) (van Dijk et al., 2009; Poitout et al., 2004), mmBCFAs exhibit anti-inflammatory properties, such as reducing necrotizing enterocolitis in neonatal rats and increasing intestinal IL-10 expression (Ran-Ressler et al., 2011), as well as suppressing IL-8 production in LPS-stimulated cells (Yan et al., 2017). Hexadecanedioic acid, a long-chain fatty acid, has been associated with antitumor activity (You et al., 2004) and may serve as a metabolic biomarker for colorectal cancer (Zhang C. et al., 2021). By contrast, eicosadienoic acid, ncommon n-6 polyunsaturated fatty acid found in human milk (Minda et al., 2004), monstrates mixed immunological effects, decreasing NO production but increasing prostaglandin E2 (PGE2) and TNF-α in LPS-stimulated macrophages (Huang et al., 2011), aligning with its harmful association in our study. Our results also showed that *RuminococcaceaeUCG011* genus*, FamilyXIII* family *and Bacteroidia* class*/Bacteroidales* order exert detrimental effects on sepsis by reducing blood levels of two glycerophospholipids, PC(34:4) (a phosphatidylcholine) and PE (P-18:0/18:1 (9Z)) (a phosphatidylethanolamine). This is supported by untargeted lipidomics studies showing that phosphatidylcholines and phosphatidylethanolamines decline in septic patients (Liang et al., 2023), and by evidence that GM-derived PE (0:0/14:0) protects against sepsis-induced intestinal injury (Tian et al., 2023). Ceramides (Cer) are well-established pro-inflammatory lipids that promote apoptosis (Tani et al., 2007) and stimulate IL-1β and other cytokines (He et al., 2016). Inhibition of ceramide accumulation can mitigate organ injury in sepsis (Peng et al., 2015), and elevated ceramide levels correlate positively with the Sequential Organ Failure Assessment (SOFA) scores in septic patients (Wu et al., 2019). Consistent with this, our MR analysis identified Cer(d18:0/18:0) as a detrimental mediator linking *Actinomycetaceae* family and *Actinomycetales* order to worse sepsis outcomes. Lysophosphatidylinositols (LysoPI), natural GPR55 agonists, have complex and sometimes conflicting roles in inflammation and immune activation (Masquelier et al., 2018; Kurano et al., 2021). Interestingly, our MR findings suggest that LysoPI(18:1 (9Z)/0:0), a mediator of *Lentisphaerae* phylum, exerts a protective effect on sepsis. Finally, trimethylamine (TMA), a well-known microbial metabolite derived from L-carnitine (Koeth et al., 2013; Rebouche et al., 1984), is preceded by the intermediate 4-trimethylammoniobutanoic acid (γ-butyrobetaine, γbb) (Koeth et al., 2014), which accumulates in the gut and is subsequently converted to TMA (Rajakovich et al., 2021). In line with this pathway, our analysis revealed that *FamilyXIII* family, *Terrisporobacter* genus, and *Sellimonas* genus can aggravate sepsis by increasing γbb levels. Pinpointing mediating blood metabolites turns a GM-sepsis association into a more testable pathway. Instead of stopping at taxa-level signals, it highlights the specific biochemical outputs through which dysbiosis may affect gut-barrier function and systemic immunity, processes widely implicated in sepsis. Practically, mediator metabolites are directly measurable in plasma, which makes them easier to validate as early risk/prognostic biomarkers and to track dynamically during critical illness. Multi-omics sepsis studies have shown that microbial and metabolic features correlate with sepsis severity/outcomes and may support early prediction and therapeutic exploration (Sun et al., 2023a). Finally, identifying which metabolic axes sit on the causal path helps prioritize actionable pathways for downstream experiments and intervention design rather than empirically manipulating taxa alone.

Our interaction network analysis identified several core targets, including IL6, STAT3, CXCL8, MMP1, MMP9, MAPK1, and MAPK3, that may play central roles in mediating GM-metabolite-sepsis interactions. IL-6 encodes a pleiotropic cytokine essential for inflammatory responses, immune regulation, tissue repair, and metabolism. IL-6, together with TNF-α and other inflammatory mediators, is a well-recognized biomarker of poor sepsis prognosis (Wu et al., 2024) and contributes to the “cytokine storm” that drives systemic inflammation and sequential organ failure in sepsis (Bösch et al., 2020; Gharamti et al., 2022). STAT3, a major downstream effector of IL-6 signaling, binds to acute phase response (APR) elements in the promoters of APR genes, thereby amplifying acute inflammatory responses (Hodge et al., 2005; Nechemia-Arbely et al., 2008). Pharmacological inhibition of STAT3 has been shown to attenuate LPS-induced acute lung injury (Zhao et al., 2016) and acute kidney injury (Zhang et al., 2017), highlighting its therapeutic relevance. CXCL8 (IL-8), absent in mice due to a genomic deletion, is a key chemokine that mediates neutrophil recruitment and activation (Yan et al., 2016), and activated leukocytes can further enhance CXCL8 production (Cui et al., 2017), creating a feed-forward inflammatory loop. MMP1 and MMP9, members of the matrix metalloproteinase (MMP) family, are critical regulators of extracellular matrix remodeling, endothelial integrity, inflammation, and coagulation during the host response to infection. However, excessive MMP activation can cause severe tissue injury and even mortality (Chen M. et al., 2022; Rodríguez et al., 2010). Plasma pro-MMP1 and active MMP1 are markedly elevated in septic patients and strongly associated with mortality, and inhibition of the murine MMP-1a ortholog improves survival, systemic inflammation, vascular permeability, and DIC in septic mice (Tressel et al., 2011). Similarly, MMP9 promotes the formation of platelet–leukocyte aggregates (PLAs), contributing to microvascular dysfunction and exacerbating sepsis severity (Chung et al., 2004; Kirschenbaum et al., 2000). Suppressing MMP9 expression reduces inflammatory responses and improves survival in septic models (Chen et al., 2020; Yao et al., 2021). Finally, MAPKs (MAPK1 and MAPK3) play a central role in inflammatory signal transduction. LPS stimulation during sepsis activates the MAPK pathway, driving enhanced release of proinflammatory cytokines (Sun et al., 2023b) and contributing to multiorgan injury (Xu et al., 2016; Alsharif et al., 2020). Inhibition of MAPK signaling confers anti-inflammatory and anti-apoptotic effects and protects against sepsis-induced liver and cardiac damage (Li et al., 2018; Zhang et al., 2024). Based on the integrated MR and network analyses, GA and 4-HPA emerged as the most promising and previously unrecognized metabolites for sepsis therapy. Thus, we selected these compounds for further experimental validation. We first demonstrated that both GA and 4-HPA significantly improved survival and reduced sepsis severity scores in LPS-induced septic mice. Sepsis frequently progresses to MODS, largely driven by an overwhelming systemic inflammatory response (Barichello et al., 2022). In line with this, we observed that GA and 4-HPA markedly reduced the serum levels of the proinflammatory cytokines TNF-α, IL-6, and IL-1β, while further elevating the anti-inflammatory cytokine IL-10. Furthermore, our molecular docking and RT-qPCR analyses suggest that GA and 4-HPA may modulate the expression of several key sepsis-related targets, including IL6, STAT3, MMP9, MMP1, MAPK1, and MAPK3, providing additional mechanistic support for their therapeutic potential.

This study has several limitations. First, the MR analyses were conducted using datasets derived primarily from individuals of European ancestry due to the limited availability of large-scale genomic data from other populations. This restricts our ability to assess racial or ethnic differences and limits the generalizability of our findings. Second, we reported nominally significant causal associations. Given the complex interplay among sepsis, the GM, and blood metabolites, and the exploratory nature of our study, applying strict multiple-testing corrections across all analyses may be overly conservative and potentially inappropriate. A stringent focus on only low FDR values could overlook biologically meaningful associations (Wang et al., 2023). Importantly, many nominally significant associations identified here are supported by previous findings, and the use of 9 MR models alongside extensive sensitivity analyses strengthens the robustness of our conclusions. Third, MR studies estimate the lifelong effects of exposures, which may not fully capture temporal or stage-specific causal relationships during sepsis progression. Nevertheless, the direction and magnitude of the inferred causal effects provide valuable insight into the potential therapeutic relevance of GM taxa and their metabolite mediators, which can be further validated through clinical studies, cellular assays, and animal experiments. Although we experimentally confirmed the protective effects of the two most promising metabolites, GA and 4-HPA, we were unable to experimentally validate all hypotheses due to limited access to certain metabolites and related reagents. The survival analysis may be underpowered because of limited follow-up duration and sample size, which could explain the lack of strong statistical significance. In addition, gut microbiota sequencing was not performed in our mouse experiments due to resource constraints; future studies integrating longitudinal microbiome profiling would strengthen the mechanistic interpretation. Moreover, we have to acknowledge that, because LD clumping at *r* ^2^ = 0.001 yielded too few instruments for several exposures, we had to use *r* ^2^ = 0.1 as the smallest feasible threshold, and thus some selected IVs may remain weakly correlated. Finally, we found that only a modest proportion of each causal effect was mediated by any single metabolite, suggesting that additional mediators likely contribute to the GM-sepsis pathway. Future studies are needed to identify and quantify these additional mediating factors. Notably, although MR strengthens causal inference by minimizing confounding and reverse causation, the assumed causal framework is inherently based on biological knowledge and methodological assumptions. Specifically, Mendelian randomization relies on three core assumptions: the genetic variants used as instrumental variables are strongly associated with the exposure of interest, are independent of potential confounders, and influence the outcome solely through the exposure without alternative pathways (Supplementary Figure S1). The causal directions specified in the present study reflect subjective judgments regarding the relationships among genetic variants, GM, metabolites, and sepsis outcomes. Moreover, the true causal structure in real-world biological systems cannot be fully verified, and the presence of unmeasured or unknown confounders, as well as complex pleiotropic pathways, cannot be completely excluded. Therefore, our findings should be interpreted as evidence supporting potential causal relationships rather than definitive proof of causality.

## 5 Conclusion

To our knowledge, this is the first study to simultaneously and comprehensively investigate the causal relationships between the GM, blood metabolites, and sepsis. Our findings highlight the critical role of blood metabolites as mediators linking GM composition to sepsis onset, progression, and outcomes. Through network pharmacology analyses, we identified key molecular targets and pathways associated with these mediator metabolites and constructed an integrated GMTS network, offering new mechanistic insights and potential therapeutic strategies for GM-related interventions in sepsis. Importantly, GA and 4-HPA emerged as pivotal metabolites within the GMTS network and demonstrated robust protective effects in experimental models of sepsis. We propose that modifying GM composition may fundamentally alter metabolism to better treat sepsis, rather than merely supplementing metabolites. Overall, our study provides genetic evidence supporting a causal GM-metabolite-sepsis axis and delivers valuable insights that may guide future mechanistic research and the development of microbiota-based therapeutic approaches for sepsis.

## Data Availability

The original contributions presented in the study are publicly available and included in the article/Supplementary Material (Sections 2.2.1, 2.3.1), further inquiries can be directed to the corresponding authors.

## References

[B1] Adelman M. W. Woodworth M. H. Langelier C. Busch L. M. Kempker J. A. Kraft C. S. (2020). The gut microbiome's role in the development, maintenance, and outcomes of sepsis. Crit. Care 24 (1), 278. 10.1186/s13054-020-02989-1 32487252 PMC7266132

[B2] Alsharif K. F. Almalki A. A. Al-Amer O. Mufti A. H. Theyab A. Lokman M. S. (2020). Oleuropein protects against lipopolysaccharide-induced sepsis and alleviates inflammatory responses in mice. IUBMB Life 72 (10), 2121–2132. 10.1002/iub.2347 32710811

[B3] Amar J. Chabo C. Waget A. Klopp P. Vachoux C. Bermúdez-Humarán L. G. (2011). Intestinal mucosal adherence and translocation of commensal bacteria at the early onset of type 2 diabetes: molecular mechanisms and probiotic treatment. EMBO Mol. Med. 3 (9), 559–572. 10.1002/emmm.201100159 21735552 PMC3265717

[B4] An S. Yao Y. Wu J. Hu H. Wu J. Sun M. (2024). Gut-derived 4-hydroxyphenylacetic acid attenuates sepsis-induced acute kidney injury by upregulating ARC to inhibit necroptosis. Biochim. Biophys. Acta Mol. Basis Dis. 1870 (1), 166876. 10.1016/j.bbadis.2023.166876 37714058

[B5] Angus D. C. van der Poll T. (2013). van der Poll T: **Sevsre sepsis and septic shock** . N. Engl. J. Med. 369 (9), 840–851. 10.1056/NEJMra1208623 23984731

[B6] Arimatsu K. Yamada H. Miyazawa H. Minagawa T. Nakajima M. Ryder M. I. (2014). Oral pathobiont induces systemic inflammation and metabolic changes associated with alteration of gut microbiota. Sci. Rep. 4, 4828. 10.1038/srep04828 24797416 PMC4010932

[B7] Azcarate-Peril M. A. Roach J. Marsh A. Chey W. D. Sandborn W. J. Ritter A. J. (2021). A double-blind, 377-subject randomized study identifies ruminococcus, coprococcus, christensenella, and collinsella as long-term potential key players in the modulation of the gut microbiome of lactose intolerant individuals by galacto-oligosaccharides. Gut Microbes 13 (1), 1957536. 10.1080/19490976.2021.1957536 34365905 PMC8354614

[B8] Bai A. Guo Y. (2017). Acid sphingomyelinase mediates human CD4(+) T-cell signaling: potential roles in T-cell responses and diseases. Cell. Death Dis. 8 (7), e2963. 10.1038/cddis.2017.360 28749465 PMC5550889

[B9] Barichello T. Generoso J. S. Singer M. Dal-Pizzol F. (2022). Biomarkers for sepsis: more than just fever and leukocytosis-a narrative review. Crit. Care 26 (1), 14. 10.1186/s13054-021-03862-5 34991675 PMC8740483

[B10] Barski O. A. Papusha V. Z. Ivanova M. M. Rudman D. M. Finegold M. J. (2005). Developmental expression and function of aldehyde reductase in proximal tubules of the kidney. Am. J. Physiol. Ren. Physiol. 289 (1), F200–F207. 10.1152/ajprenal.00411.2004 15769935

[B11] Bösch F. Schallhorn S. Miksch R. C. Chaudry I. H. Faist E. Werner J. (2020). The prognostic value of presepsin for sepsis in abdominal surgery: a prospective study. Shock 54 (1), 56–61. 10.1097/SHK.0000000000001479 31743301

[B12] Bowden J. Holmes M. V. (2019). Meta-analysis and Mendelian randomization: a review. Res. Synth. Methods 10 (4), 486–496. 10.1002/jrsm.1346 30861319 PMC6973275

[B13] Bowden J. Davey Smith G. Burgess S. (2015). Mendelian randomization with invalid instruments: effect estimation and bias detection through egger regression. Int. J. Epidemiol. 44 (2), 512–525. 10.1093/ije/dyv080 26050253 PMC4469799

[B14] Bowden J. Davey Smith G. Haycock P. C. Burgess S. (2016). Consistent estimation in Mendelian randomization with some invalid instruments using a weighted median estimator. Genet. Epidemiol. 40 (4), 304–314. 10.1002/gepi.21965 27061298 PMC4849733

[B15] Burgess S. (2014). Sample size and power calculations in Mendelian randomization with a single instrumental variable and a binary outcome. Int. J. Epidemiol. 43 (3), 922–929. 10.1093/ije/dyu005 24608958 PMC4052137

[B16] Burgess S. Thompson S. G. CRP CHD Genetics Collaboration (2011). Avoiding bias from weak instruments in Mendelian randomization studies. Int. J. Epidemiol. 40 (3), 755–764. 10.1093/ije/dyr036 21414999

[B17] Burgess S. Dudbridge F. Thompson S. G. (2016). Combining information on multiple instrumental variables in Mendelian randomization: comparison of allele score and summarized data methods. Stat. Med. 35 (11), 1880–1906. 10.1002/sim.6835 26661904 PMC4832315

[B18] Bycroft C. Freeman C. Petkova D. Band G. Elliott L. T. Sharp K. (2018). The UK biobank resource with deep phenotyping and genomic data. Nature 562 (7726), 203–209. 10.1038/s41586-018-0579-z 30305743 PMC6786975

[B19] Chen Z. Wang S. Chen Y. Shao Z. Yu Z. Mei S. (2020). Integrin β3 modulates TLR4-Mediated inflammation by regulation of CD14 expression in macrophages in septic condition. Shock 53 (3), 335–343. 10.1097/SHK.0000000000001383 31135705

[B20] Chen Q. Liang X. Wu T. Jiang J. Jiang Y. Zhang S. (2022a). Integrative analysis of metabolomics and proteomics reveals amino acid metabolism disorder in sepsis. J. Transl. Med. 20 (1), 123. 10.1186/s12967-022-03320-y 35287674 PMC8919526

[B21] Chen M. Su W. Chen F. Lai T. Liu Y. Yu D. (2022b). Mechanisms underlying the therapeutic effects of 4-octyl itaconate in treating sepsis based on network pharmacology and molecular docking. Front. Genet. 13, 1056405. 10.3389/fgene.2022.1056405 36406124 PMC9671214

[B22] Chen Z. Chen Z. Jin X. (2023a). Mendelian randomization supports causality between overweight status and accelerated aging. Aging Cell. 22 (8), e13899. 10.1111/acel.13899 37277933 PMC10410004

[B23] Chen Y. Lu T. Pettersson-Kymmer U. Stewart I. D. Butler-Laporte G. Nakanishi T. (2023b). Genomic atlas of the plasma metabolome prioritizes metabolites implicated in human diseases. Nat. Genet. 55 (1), 44–53. 10.1038/s41588-022-01270-1 36635386 PMC7614162

[B24] Chen Y. Xie Y. Ci H. Cheng Z. Kuang Y. Li S. (2024a). Plasma metabolites and risk of seven cancers: a two-sample Mendelian randomization study among European descendants. BMC Med. 22 (1), 90. 10.1186/s12916-024-03272-8 38433226 PMC10910673

[B25] Chen K. Wang D. Qian M. Weng M. Lu Z. Zhang K. (2024b). Endothelial cell dysfunction and targeted therapeutic drugs in sepsis. Heliyon 10 (13), e33340. 10.1016/j.heliyon.2024.e33340 39027563 PMC11255673

[B26] Chung A. W. Radomski A. Alonso-Escolano D. Jurasz P. Stewart M. W. Malinski T. (2004). Platelet-leukocyte aggregation induced by PAR agonists: regulation by nitric oxide and matrix metalloproteinases. Br. J. Pharmacol. 143 (7), 845–855. 10.1038/sj.bjp.0705997 15533889 PMC1575938

[B27] Cui S. Zhu Y. Du J. Khan M. N. Wang B. Wei J. (2017). CXCL8 antagonist improves diabetic nephropathy in Male mice with diabetes and attenuates high glucose-induced mesangial injury. Endocrinology 158 (6), 1671–1684. 10.1210/en.2016-1781 28387853

[B28] Daina A. Zoete V. (2024). Testing the predictive power of reverse screening to infer drug targets, with the help of machine learning. Commun. Chemistry 7 (1), 105. 10.1038/s42004-024-01179-2 38724725 PMC11082207

[B29] Dao L. Liu H. Xiu R. Yao T. Tong R. Xu L. (2024). Gramine improves sepsis-induced myocardial dysfunction by binding to NF-κB p105 and inhibiting its ubiquitination. Phytomedicine 125, 155325. 10.1016/j.phymed.2023.155325 38295663

[B30] Denburg M. R. Xu Y. Abraham A. G. Coresh J. Chen J. Grams M. E. (2021). Metabolite biomarkers of CKD progression in children. Clin. J. Am. Soc. Nephrol. 16 (8), 1178–1189. 10.2215/CJN.00220121 34362785 PMC8455058

[B31] Diener C. Dai C. L. Wilmanski T. Baloni P. Smith B. Rappaport N. (2022). Genome-microbiome interplay provides insight into the determinants of the human blood metabolome. Nat. Metab. 4 (11), 1560–1572. 10.1038/s42255-022-00670-1 36357685 PMC9691620

[B32] Eberhardt J. Santos-Martins D. Tillack A. F. Forli S. (2021). AutoDock vina 1.2.0: new docking methods, expanded force field, and python bindings. J. Chem. Inf. Model. 61 (8), 3891–3898. 10.1021/acs.jcim.1c00203 34278794 PMC10683950

[B33] Formosa A. Turgeon P. dos Santos C. C. (2022). Role of miRNA dysregulation in sepsis. Mol. Med. 28 (1), 99. 10.1186/s10020-022-00527-z 35986237 PMC9389495

[B34] Gai X. Wang H. Li Y. Zhao H. He C. Wang Z. (2021). Fecal microbiota transplantation protects the intestinal mucosal barrier by reconstructing the gut microbiota in a murine model of sepsis. Front. Cell. Infect. Microbiol. 11, 736204. 10.3389/fcimb.2021.736204 34631604 PMC8493958

[B35] Gharamti A. A. Samara O. Monzon A. Montalbano G. Scherger S. DeSanto K. (2022). Proinflammatory cytokines levels in sepsis and healthy volunteers, and tumor necrosis factor-alpha associated sepsis mortality: a systematic review and meta-analysis. Cytokine 158, 156006. 10.1016/j.cyto.2022.156006 36044827

[B36] Ghosh A. Nishtala K. (2017). Biofluid lipidome: a source for potential diagnostic biomarkers. Clin. Transl. Med. 6 (1), 22. 10.1186/s40169-017-0152-7 28639235 PMC5479868

[B37] Haak B. W. Wiersinga W. J. (2017). The role of the gut microbiota in sepsis. Lancet Gastroenterol. Hepatol. 2 (2), 135–143. 10.1016/S2468-1253(16)30119-4 28403983

[B38] Hartwig F. P. Davey Smith G. Bowden J. (2017). Robust inference in summary data Mendelian randomization *via* the zero modal pleiotropy assumption. Int. J. Epidemiol. 46 (6), 1985–1998. 10.1093/ije/dyx102 29040600 PMC5837715

[B39] He Y. Hara H. Núñez G. (2016). Mechanism and regulation of NLRP3 inflammasome activation. Trends Biochem. Sci. 41 (12), 1012–1021. 10.1016/j.tibs.2016.09.002 27669650 PMC5123939

[B40] He S. Lin F. Hu X. Pan P. (2023). Gut microbiome-based therapeutics in critically ill adult Patients-A narrative review. Nutrients 15 (22), 4734. 10.3390/nu15224734 38004128 PMC10675331

[B41] Hodge D. R. Hurt E. M. Farrar W. L. (2005). The role of IL-6 and STAT3 in inflammation and cancer. Eur. J. Cancer 41 (16), 2502–2512. 10.1016/j.ejca.2005.08.016 16199153

[B42] Hotchkiss R. S. Moldawer L. L. Opal S. M. Reinhart K. Turnbull I. R. Vincent J.-L. (2016). Sepsis and septic shock. Nat. Rev. Dis. Prim. 2 (1), 16045. 10.1038/nrdp.2016.45 28117397 PMC5538252

[B43] Hsin K. Y. Ghosh S. Kitano H. (2013). Combining machine learning systems and multiple docking simulation packages to improve docking prediction reliability for network pharmacology. PLoS One 8 (12), e83922. 10.1371/journal.pone.0083922 24391846 PMC3877102

[B44] Huang S. (2023). Efficient analysis of toxicity and mechanisms of environmental pollutants with network toxicology and molecular docking strategy: acetyl tributyl citrate as an example. Sci. Total Environ. 905, 167904. 10.1016/j.scitotenv.2023.167904 37858827

[B45] Huang Y. S. Huang W. C. Li C. W. Chuang L. T. (2011). Eicosadienoic acid differentially modulates production of pro-inflammatory modulators in murine macrophages. Mol. Cell. Biochem. 358 (1-2), 85–94. 10.1007/s11010-011-0924-0 21688154

[B46] Huang Z. B. Hu Z. Lu C. X. Luo S. D. Chen Y. Zhou Z. P. (2022). Gut microbiota-derived indole 3-propionic acid partially activates aryl hydrocarbon receptor to promote macrophage phagocytosis and attenuate septic injury. Front. Cell. Infect. Microbiol. 12, 1015386. 10.3389/fcimb.2022.1015386 36299625 PMC9589056

[B47] Jaurila H. Koivukangas V. Koskela M. Gäddnäs F. Myllymaa S. Kullaa A. (2020). (1)H NMR based metabolomics in human sepsis and healthy serum. Metabolites 10 (2). 10.3390/metabo10020070 32075299 PMC7074315

[B48] Jenner A. M. Rafter J. Halliwell B. (2005). Human fecal water content of phenolics: the extent of colonic exposure to aromatic compounds. Free Radic. Biol. Med. 38 (6), 763–772. 10.1016/j.freeradbiomed.2004.11.020 15721987

[B49] Ji L. Song T. Ge C. Wu Q. Ma L. Chen X. (2023). Identification of bioactive compounds and potential mechanisms of scutellariae radix-coptidis rhizoma in the treatment of atherosclerosis by integrating network pharmacology and experimental validation. Biomed. Pharmacother. 165, 115210. 10.1016/j.biopha.2023.115210 37499457

[B50] Kamada N. Seo S. U. Chen G. Y. Núñez G. (2013). Role of the gut microbiota in immunity and inflammatory disease. Nat. Rev. Immunol. 13 (5), 321–335. 10.1038/nri3430 23618829

[B51] Kamat M. A. Blackshaw J. A. Young R. Surendran P. Burgess S. Danesh J. (2019). PhenoScanner V2: an expanded tool for searching human genotype-phenotype associations. Bioinformatics 35 (22), 4851–4853. 10.1093/bioinformatics/btz469 31233103 PMC6853652

[B52] Kim D. H. Jung E. A. Sohng I. S. Han J. A. Kim T. H. Han M. J. (1998). Intestinal bacterial metabolism of flavonoids and its relation to some biological activities. Arch. Pharm. Res. 21 (1), 17–23. 10.1007/BF03216747 9875509

[B53] Kirschenbaum L. A. Aziz M. Astiz M. E. Saha D. C. Rackow E. C. (2000). Influence of rheologic changes and platelet-neutrophil interactions on cell filtration in sepsis. Am. J. Respir. Crit. Care Med. 161 (5), 1602–1607. 10.1164/ajrccm.161.5.9902105 10806162

[B54] Kniazeva M. Crawford Q. T. Seiber M. Wang C. Y. Han M. (2004). Monomethyl branched-chain fatty acids play an essential role in *Caenorhabditis elegans* development. PLoS Biol. 2 (9), E257. 10.1371/journal.pbio.0020257 15340492 PMC514883

[B55] Koeth R. A. Wang Z. Levison B. S. Buffa J. A. Org E. Sheehy B. T. (2013). Intestinal microbiota metabolism of L-carnitine, a nutrient in red meat, promotes atherosclerosis. Nat. Med. 19 (5), 576–585. 10.1038/nm.3145 23563705 PMC3650111

[B56] Koeth R. A. Levison B. S. Culley M. K. Buffa J. A. Wang Z. Gregory J. C. (2014). γ-Butyrobetaine is a proatherogenic intermediate in gut microbial metabolism of L-carnitine to TMAO. Cell. Metab. 20 (5), 799–812. 10.1016/j.cmet.2014.10.006 25440057 PMC4255476

[B57] Kolde R. Laur S. Adler P. Vilo J. (2012). Robust rank aggregation for gene list integration and meta-analysis. Bioinformatics 28 (4), 573–580. 10.1093/bioinformatics/btr709 22247279 PMC3278763

[B58] Kumari S. Deori M. Elancheran R. Kotoky J. Devi R. (2016). *In vitro* and *in vivo* antioxidant, anti-hyperlipidemic properties and chemical characterization of *Centella asiatica* (L.) extract. Front. Pharmacol. 7, 400. 10.3389/fphar.2016.00400 27840607 PMC5083837

[B59] Kumari S. Elancheran R. Devi R. (2018). Phytochemical screening, antioxidant, antityrosinase, and antigenotoxic potential of Amaranthus viridis extract. Indian J. Pharmacol. 50 (3), 130–138. 10.4103/ijp.IJP_77_18 30166750 PMC6106121

[B60] Kurano M. Kobayashi T. Sakai E. Tsukamoto K. Yatomi Y. (2021). Lysophosphatidylinositol, especially albumin-bound form, induces inflammatory cytokines in macrophages. Faseb J. 35 (6), e21673. 10.1096/fj.202100245R 34042213

[B61] Kurilshikov A. Medina-Gomez C. Bacigalupe R. Radjabzadeh D. Wang J. Demirkan A. (2021). Large-scale association analyses identify host factors influencing human gut microbiome composition. Nat. Genet. 53 (2), 156–165. 10.1038/s41588-020-00763-1 33462485 PMC8515199

[B62] Li P. Chen X. R. Xu F. Liu C. Li C. Liu H. (2018). Alamandine attenuates sepsis-associated cardiac dysfunction *via* inhibiting MAPKs signaling pathways. Life Sci. 206, 106–116. 10.1016/j.lfs.2018.04.010 29679702

[B63] Li R. Guo C. Li Y. Qin Z. Huang W. (2021). Therapeutic targets and signaling mechanisms of vitamin C activity against sepsis: a bioinformatics study. Brief. Bioinform 22 (3), bbaa079. 10.1093/bib/bbaa079 32393985 PMC7454291

[B64] Li F. Jiang L. Pan S. Jiang S. Fan Y. Jiang C. (2022). Multi-omic profiling reveals that intra-abdominal-hypertension-induced intestinal damage can be prevented by microbiome and metabolic modulations with 5-Hydroxyindoleacetic acid as a diagnostic marker. mSystems 7 (3), e0120421. 10.1128/msystems.01204-21 35574681 PMC9238425

[B65] Li J. Chen Y. Li R. Zhang X. Chen T. Mei F. (2023). Gut microbial metabolite hyodeoxycholic acid targets the TLR4/MD2 complex to attenuate inflammation and protect against sepsis. Mol. Ther. 31 (4), 1017–1032. 10.1016/j.ymthe.2023.01.018 36698311 PMC10124078

[B66] Liang J. Li J. Zhang J. Rong J. Wang X. Zhao C. (2023). UHPLC-MS/MS-based untargeted lipidomics analysis of septic patients. Clin. Chim. Acta 544, 117336. 10.1016/j.cca.2023.117336 37031781

[B67] Liu Z. Xi R. Zhang Z. Li W. Liu Y. Jin F. (2014). 4-hydroxyphenylacetic acid attenuated inflammation and edema *via* suppressing HIF-1α in seawater aspiration-induced lung injury in rats. Int. J. Mol. Sci. 15 (7), 12861–12884. 10.3390/ijms150712861 25050781 PMC4139878

[B68] Liu Z. Li N. Fang H. Chen X. Guo Y. Gong S. (2019). Enteric dysbiosis is associated with sepsis in patients. Faseb J. 33 (11), 12299–12310. 10.1096/fj.201900398RR 31465241 PMC6902702

[B69] Liu X. Tong X. Zou Y. Lin X. Zhao H. Tian L. (2022). Mendelian randomization analyses support causal relationships between blood metabolites and the gut microbiome. Nat. Genet. 54 (1), 52–61. 10.1038/s41588-021-00968-y 34980918

[B70] Lv Y. Cheng X. Dong Q. (2024). SGLT1 and SGLT2 inhibition, circulating metabolites, and cerebral small vessel disease: a mediation mendelian randomization study. Cardiovasc Diabetol. 23 (1), 157. 10.1186/s12933-024-02255-6 38715111 PMC11077823

[B71] Marshall J. C. (2014). Why have clinical trials in sepsis failed? Trends Mol. Med. 20 (4), 195–203. 10.1016/j.molmed.2014.01.007 24581450

[B72] Masquelier J. Alhouayek M. Terrasi R. Bottemanne P. Paquot A. Muccioli G. G. (2018). Lysophosphatidylinositols in inflammation and macrophage activation: altered levels and anti-inflammatory effects. Biochim. Biophys. Acta Mol. Cell. Biol. Lipids 1863 (12), 1458–1468. 10.1016/j.bbalip.2018.09.003 30251703

[B73] Minda H. Kovács A. Funke S. Szász M. Burus I. Molnár S. (2004). Changes of fatty acid composition of human milk during the first month of lactation: a day-to-day approach in the first week. Ann. Nutr. Metab. 48 (3), 202–209. 10.1159/000079821 15256803

[B74] Mu S. Xiang H. Wang Y. Wei W. Long X. Han Y. (2022). The pathogens of secondary infection in septic patients share a similar genotype to those that predominate in the gut. Crit. Care 26 (1), 68. 10.1186/s13054-022-03943-z 35331299 PMC8944137

[B75] Mukhopadhya I. Hansen R. El-Omar E. M. Hold G. L. (2012). IBD-What role do proteobacteria play? Nat. Rev. Gastroenterol. Hepatol. 9 (4), 219–230. 10.1038/nrgastro.2012.14 22349170

[B76] Muratsu A. Ikeda M. Shimizu K. Kameoka S. Motooka D. Nakamura S. (2022). Dynamic change of fecal microbiota and metabolomics in a polymicrobial murine sepsis model. Acute Med. Surg. 9 (1), e770. 10.1002/ams2.770 35782956 PMC9238289

[B77] Napolitano F. Giudice V. Selleri C. Montuori N. (2023). Plasminogen system in the pathophysiology of sepsis: upcoming biomarkers. Int. J. Mol. Sci. 24 (15), 12376. 10.3390/ijms241512376 37569751 PMC10418678

[B78] Nechemia-Arbely Y. Barkan D. Pizov G. Shriki A. Rose-John S. Galun E. (2008). IL-6/IL-6R axis plays a critical role in acute kidney injury. J. Am. Soc. Nephrol. 19 (6), 1106–1115. 10.1681/ASN.2007070744 18337485 PMC2396933

[B79] Nickel J. Gohlke B. O. Erehman J. Banerjee P. Rong W. W. Goede A. (2014). SuperPred: update on drug classification and target prediction. Nucleic Acids Research 42 (Web Server issue), W26–W31. 10.1093/nar/gku477 24878925 PMC4086135

[B80] Nüse B. Holland T. Rauh M. Gerlach R. G. Mattner J. (2023). L-arginine metabolism as pivotal interface of mutual host-microbe interactions in the gut. Gut Microbes 15 (1), 2222961. 10.1080/19490976.2023.2222961 37358082 PMC10294761

[B81] O'Keefe S. J. Ou J. Delany J. P. Curry S. Zoetendal E. Gaskins H. R. (2011). Effect of fiber supplementation on the microbiota in critically ill patients. World J. Gastrointest. Pathophysiol. 2 (6), 138–145. 10.4291/wjgp.v2.i6.138 22180847 PMC3240905

[B82] Oh K. K. Choi Y. R. Gupta H. Ganesan R. Sharma S. P. Won S. M. (2022). Identification of gut microbiome metabolites *via* network pharmacology analysis in treating alcoholic liver disease. Curr. Issues Mol. Biol. 44 (7), 3253–3266. 10.3390/cimb44070224 35877448 PMC9316215

[B83] Oh K. K. Gupta H. Min B. H. Ganesan R. Sharma S. P. Won S. M. (2023a). The identification of metabolites from gut microbiota in NAFLD *via* network pharmacology. Sci. Rep. 13 (1), 724. 10.1038/s41598-023-27885-w 36639568 PMC9839744

[B84] Oh K. K. Yoon S. J. Lee S. B. Lee S. Y. Gupta H. Ganesan R. (2023b). The convergent application of metabolites from Avena sativa and gut microbiota to ameliorate non-alcoholic fatty liver disease: a network pharmacology study. J. Transl. Med. 21 (1), 263. 10.1186/s12967-023-04122-6 37069607 PMC10111676

[B85] Park D. W. Kwak D. S. Park Y. Y. Chang Y. Huh J. W. Lim C. M. (2014). Impact of serial measurements of lysophosphatidylcholine on 28-day mortality prediction in patients admitted to the intensive care unit with severe sepsis or septic shock. J. Crit. Care 29 (5), 882–885. 10.1016/j.jcrc.2014.05.003 24961965

[B86] Peng H. Li C. Kadow S. Henry B. D. Steinmann J. Becker K. A. (2015). Acid sphingomyelinase inhibition protects mice from lung edema and lethal *Staphylococcus aureus* sepsis. J. Mol. Med. Berl. 93 (6), 675–689. 10.1007/s00109-014-1246-y 25616357 PMC4432103

[B87] Peng Y. Wei J. Jia X. Luan F. Man M. Ma X. (2022). Changes in the microbiota in different intestinal segments of mice with sepsis. Front. Cell. Infect. Microbiol. 12, 954347. 10.3389/fcimb.2022.954347 36704101 PMC9871835

[B88] Pierce B. L. Burgess S. (2013). Efficient design for Mendelian randomization studies: subsample and 2-sample instrumental variable estimators. Am. J. Epidemiol. 178 (7), 1177–1184. 10.1093/aje/kwt084 23863760 PMC3783091

[B89] Płóciennikowska A. Hromada-Judycka A. Borzęcka K. Kwiatkowska K. (2015). Co-operation of TLR4 and raft proteins in LPS-Induced pro-inflammatory signaling. Cell. Mol. Life Sci. 72 (3), 557–581. 25332099 10.1007/s00018-014-1762-5 PMC4293489

[B90] Poitout V. Briaud I. Kelpe C. Hagman D. (2004). Gluco-lipotoxicity of the pancreatic beta cell. Ann. Endocrinol. Paris. 65 (1), 37–41. 10.1016/s0003-4266(04)95628-4 15122090

[B91] Rajakovich L. J. Fu B. Bollenbach M. Balskus E. P. (2021). Elucidation of an anaerobic pathway for metabolism of l-carnitine-derived γ-butyrobetaine to trimethylamine in human gut bacteria. Proc. Natl. Acad. Sci. U. S. A. 118 (32), e2101498118. 10.1073/pnas.2101498118 34362844 PMC8364193

[B92] Rajilić-Stojanović M. de Vos W. M. (2014). The first 1000 cultured species of the human gastrointestinal microbiota. FEMS Microbiol. Rev. 38 (5), 996–1047. 10.1111/1574-6976.12075 24861948 PMC4262072

[B93] Ran-Ressler R. R. Khailova L. Arganbright K. M. Adkins-Rieck C. K. Jouni Z. E. Koren O. (2011). Branched chain fatty acids reduce the incidence of necrotizing enterocolitis and alter gastrointestinal microbial ecology in a neonatal rat model. PLoS One 6 (12), e29032. 10.1371/journal.pone.0029032 22194981 PMC3237582

[B94] Rebouche C. J. Mack D. L. Edmonson P. F. (1984). L-Carnitine dissimilation in the gastrointestinal tract of the rat. Biochemistry 23 (26), 6422–6426. 10.1021/bi00321a022 6529558

[B95] Rival T. Cinq-Frais C. Silva-Sifontes S. Garcia J. Riu B. Salvayre R. (2013). Alteration of plasma phospholipid fatty acid profile in patients with septic shock. Biochimie 95 (11), 2177–2181. 10.1016/j.biochi.2013.08.006 23954620

[B96] Rodríguez D. Morrison C. J. Overall C. M. (2010). Matrix metalloproteinases: what do they not do? New substrates and biological roles identified by murine models and proteomics. Biochim. Biophys. Acta 1803 (1), 39–54. 19800373 10.1016/j.bbamcr.2009.09.015

[B97] Rudd K. E. Johnson S. C. Agesa K. M. Shackelford K. A. Tsoi D. Kievlan D. R. (2020). Global, regional, and national sepsis incidence and mortality, 1990-2017: analysis for the global burden of disease study. Lancet 395 (10219), 200–211. 10.1016/S0140-6736(19)32989-7 31954465 PMC6970225

[B98] Shang W. Zhang S. Qian H. Huang S. Li H. Liu J. (2024). Gut microbiota and sepsis and sepsis-related death: a Mendelian randomization investigation. Front. Immunol. 15, 1266230. 10.3389/fimmu.2024.1266230 38361921 PMC10867964

[B99] Shen Y. Xu J. Li Z. Huang Y. Yuan Y. Wang J. (2018). Analysis of gut microbiota diversity and auxiliary diagnosis as a biomarker in patients with schizophrenia: a cross-sectional study. Schizophr. Res. 197, 470–477. 10.1016/j.schres.2018.01.002 29352709

[B100] Shrum B. Anantha R. V. Xu S. X. Donnelly M. Haeryfar S. M. McCormick J. K. (2014). A robust scoring system to evaluate sepsis severity in an animal model. BMC Res. Notes 7, 233. 10.1186/1756-0500-7-233 24725742 PMC4022086

[B101] Skrivankova V. W. Richmond R. C. Woolf B. A. R. Yarmolinsky J. Davies N. M. Swanson S. A. (2021). Strengthening the reporting of observational studies in epidemiology using mendelian randomization: the STROBE-MR statement. Jama 326 (16), 1614–1621. 10.1001/jama.2021.18236 34698778

[B102] Smith G. D. Ebrahim S. (2003). Mendelian randomization': can genetic epidemiology contribute to understanding environmental determinants of disease? Int. J. Epidemiol. 32 (1), 1–22. 10.1093/ije/dyg070 12689998

[B103] Stoma I. Littmann E. R. Peled J. U. Giralt S. van den Brink M. R. M. Pamer E. G. (2021). Compositional flux within the intestinal microbiota and risk for bloodstream infection with gram-negative bacteria. Clin. Infect. Dis. 73 (11), e4627–e4635. 10.1093/cid/ciaa068 31976518 PMC8662789

[B104] Su L. Huang Y. Zhu Y. Xia L. Wang R. Xiao K. (2014). Discrimination of sepsis stage metabolic profiles with an LC/MS-MS-based metabolomics approach. BMJ Open Respir. Res. 1 (1), e000056. 10.1136/bmjresp-2014-000056 25553245 PMC4265126

[B105] Sun D. Bai R. Zhou W. Yao Z. Liu Y. Tang S. (2021). Angiogenin maintains gut microbe homeostasis by balancing α-Proteobacteria and lachnospiraceae. Gut 70 (4), 666–676. 10.1136/gutjnl-2019-320135 32843357 PMC7904960

[B106] Sun S. Wang D. Dong D. Xu L. Xie M. Wang Y. (2023a). Altered intestinal microbiome and metabolome correspond to the clinical outcome of sepsis. Crit. Care 27 (1), 127. 10.1186/s13054-023-04412-x 36978107 PMC10044080

[B107] Sun S. Wang L. Wang J. Chen R. Pei S. Yao S. (2023b). Maresin1 prevents sepsis-induced acute liver injury by suppressing NF-κB/Stat3/MAPK pathways, mitigating inflammation. Heliyon 9 (11), e21883. 10.1016/j.heliyon.2023.e21883 38027581 PMC10665730

[B108] Szabó B. G. Kiss R. Makra N. Pénzes K. Vad E. Kamotsay K. (2022). Composition and changes of blood microbiota in adult patients with community-acquired sepsis: a pilot study from bench to bedside. Front. Cell. Infect. Microbiol. 12, 1067476. 10.3389/fcimb.2022.1067476 36583109 PMC9794134

[B109] Tani M. Ito M. Igarashi Y. (2007). Ceramide/Sphingosine/Sphingosine 1-phosphate metabolism on the cell surface and in the extracellular space. Cell. Signal 19 (2), 229–237. 10.1016/j.cellsig.2006.07.001 16963225

[B110] Tian W. Z. Yue Q. Fei W. Yao P. Z. Han R. Q. Tang J. (2023). PE (0:0/14:0), an endogenous metabolite of the gut microbiota, exerts protective effects against sepsis-induced intestinal injury by modulating the AHR/CYP1A1 pathway. Clin. Sci. (Lond) 137 (22), 1753–1769. 10.1042/CS20230704 37921121

[B111] Tofighi D. MacKinnon D. P. (2011). RMediation: an R package for mediation analysis confidence intervals. Behav. Res. Methods 43 (3), 692–700. 10.3758/s13428-011-0076-x 21487904 PMC3233842

[B112] Tressel S. L. Kaneider N. C. Kasuda S. Foley C. Koukos G. Austin K. (2011). A matrix metalloprotease-PAR1 system regulates vascular integrity, systemic inflammation and death in sepsis. EMBO Mol. Med. 3 (7), 370–384. 10.1002/emmm.201100145 21591259 PMC3394510

[B113] van Dijk S. J. Feskens E. J. Bos M. B. Hoelen D. W. Heijligenberg R. Bromhaar M. G. (2009). A saturated fatty acid-rich diet induces an obesity-linked proinflammatory gene expression profile in adipose tissue of subjects at risk of metabolic syndrome. Am. J. Clin. Nutr. 90 (6), 1656–1664. 10.3945/ajcn.2009.27792 19828712

[B114] Verbanck M. Chen C. Y. Neale B. Do R. (2018). Detection of widespread horizontal pleiotropy in causal relationships inferred from Mendelian randomization between complex traits and diseases. Nat. Genet. 50 (5), 693–698. 10.1038/s41588-018-0099-7 29686387 PMC6083837

[B115] Vissiennon C. Nieber K. Kelber O. Butterweck V. (2012). Route of administration determines the anxiolytic activity of the flavonols kaempferol, quercetin and myricetin--are they prodrugs? J. Nutr. Biochem. 23 (7), 733–740. 10.1016/j.jnutbio.2011.03.017 21840194

[B116] Wang X. Shen Y. Wang S. Li S. Zhang W. Liu X. (2017). PharmMapper 2017 update: a web server for potential drug target identification with a comprehensive target pharmacophore database. Nucleic Acids Research 45 (W1), W356–w360. 10.1093/nar/gkx374 28472422 PMC5793840

[B117] Wang Q. Dai H. Hou T. Hou Y. Wang T. Lin H. (2023). Dissecting causal relationships between gut microbiota, blood metabolites, and stroke: a mendelian randomization study. J. Stroke 25 (3), 350–360. 10.5853/jos.2023.00381 37813672 PMC10574297

[B118] Wei Z. Xiong Q. Huang D. Wu Z. Chen Z. (2023). Causal relationship between blood metabolites and risk of five infections: a Mendelian randomization study. BMC Infect. Dis. 23 (1), 663. 10.1186/s12879-023-08662-6 37805474 PMC10559484

[B119] Wong J. Piceno Y. M. DeSantis T. Z. Pahl M. Andersen G. L. Vaziri N. D. (2014). Expansion of urease- and uricase-containing, indole- and p-cresol-forming and contraction of short-chain fatty acid-producing intestinal microbiota in ESRD. Am. J. Nephrol. 39 (3), 230–237. 10.1159/000360010 24643131 PMC4049264

[B120] Wu X. Hou J. Li H. Xie G. Zhang X. Zheng J. (2019). Inverse correlation between plasma Sphingosine-1-Phosphate and ceramide concentrations in septic patients and their utility in predicting mortality. Shock 51 (6), 718–724. 10.1097/SHK.0000000000001229 30080743 PMC6511430

[B121] Wu H. Jia S. Liao B. Ji T. Huang J. Luo Y. (2024). Establishment of a mortality risk nomogram for predicting in-hospital mortality of sepsis: cohort study from a Chinese single center. Front. Med. (Lausanne) 11, 1360197. 10.3389/fmed.2024.1360197 38765257 PMC11100418

[B122] Xie S. Li J. Lyu F. Xiong Q. Gu P. Chen Y. (2023). Novel tripeptide RKH derived from Akkermansia muciniphila protects against lethal sepsis. Gut 73 (1), 78–91. 10.1136/gutjnl-2023-329996 37553229

[B123] Xu J. Wang K. Q. Xu W. H. Li Y. H. Qi Y. Wu H. Y. (2016). The matrine derivate MASM prolongs survival, attenuates inflammation, and reduces organ injury in murine established lethal sepsis. J. Infect. Dis. 214 (11), 1762–1772. 10.1093/infdis/jiw445 27658692

[B124] Yan W. Li F. Qin Y. Ren Y. Zheng L. Dai X. (2016). Evaluation of recombinant CXCL8(3-73)K11R/G31P in muscle fibrosis and Trichinella larvae encapsulation in a murine model of trichinellosis. Int. Immunopharmacol. 35, 323–326. 10.1016/j.intimp.2016.03.047 27089392

[B125] Yan Y. Wang Z. Greenwald J. Kothapalli K. S. Park H. G. Liu R. (2017). BCFA suppresses LPS induced IL-8 mRNA expression in human intestinal epithelial cells. Prostagl. Leukot. Essent. Fat. Acids 116, 27–31. 10.1016/j.plefa.2016.12.001 28088291

[B126] Yang R. Shan S. Shi J. Li H. An N. Li S. (2023). Coprococcus eutactus, a potent probiotic, alleviates colitis *via* acetate-mediated IgA response and microbiota restoration. J. Agric. Food Chem. 10.1021/acs.jafc.2c06697 36786768

[B127] Yao Z. J. Dong J. Che Y. J. Zhu M. F. Wen M. Wang N. N. (2016). TargetNet: a web service for predicting potential drug-target interaction profiling *via* multi-target SAR models. J. Computer-Aided Molecular Design 30 (5), 413–424. 10.1007/s10822-016-9915-2 27167132

[B128] Yao T. Zhang L. Fu Y. Yao L. Zhou C. Chen G. (2021). Saikosaponin-d alleviates renal inflammation and cell apoptosis in a mouse model of sepsis *via* TCF7/FOSL1/Matrix metalloproteinase 9 inhibition. Mol. Cell. Biol. 41 (10), e0033221. 10.1128/MCB.00332-21 34309413 PMC8462463

[B129] You Y. J. Kim Y. Nam N. H. Bang S. C. Ahn B. Z. (2004). Alkyl and carboxylalkyl esters of 4'-demethyl-4-deoxypodophyllotoxin: synthesis, cytotoxic, and antitumor activity. Eur. J. Med. Chem. 39 (2), 189–193. 10.1016/j.ejmech.2003.10.002 14987827

[B130] Yu C. Zhu X. Zheng C. Luo Y. Wang F. Gao Y. (2021). Methyl diet enhanced sepsis-induced mortality through altering gut microbiota. J. Inflamm. Res. 14, 3107–3121. 10.2147/JIR.S305202 34276224 PMC8277458

[B131] Yu J. Li H. Zhao J. Huang Y. Liu C. Yang P. (2022). Alterations of the gut microbiome in Chinese Zhuang ethnic patients with sepsis. Mediat. Inflamm. 2022, 2808249. 10.1155/2022/2808249 35633656 PMC9142305

[B132] Zhang Y. Sanner M. F. (2019). AutoDock CrankPep: combining folding and docking to predict protein-peptide complexes. Bioinformatics 35 (24), 5121–5127. 10.1093/bioinformatics/btz459 31161213 PMC6954657

[B133] Zhang S. Ma J. Sheng L. Zhang D. Chen X. Yang J. (2017). Total coumarins from Hydrangea paniculata show renal protective effects in lipopolysaccharide-induced acute kidney injury *via* anti-inflammatory and antioxidant activities. Front. Pharmacol. 8, 872. 10.3389/fphar.2017.00872 29311915 PMC5735979

[B134] Zhang H. Zhuo S. Song D. Wang L. Gu J. Ma J. (2021a). Icariin inhibits intestinal inflammation of DSS-induced colitis mice through modulating intestinal flora abundance and modulating p-p65/p65 molecule. Turk J. Gastroenterol. 32 (4), 382–392. 10.5152/tjg.2021.20282 34231485 PMC8975518

[B135] Zhang C. Zhou S. Chang H. Zhuang F. Shi Y. Chang L. (2021b). Metabolomic profiling identified serum metabolite biomarkers and related metabolic pathways of colorectal cancer. Dis. Markers 2021, 6858809. 10.1155/2021/6858809 34917201 PMC8670981

[B136] Zhang L. Zhang T. Sun J. Huang Y. Liu T. Ye Z. (2023). Calorie restriction ameliorates hyperglycemia, modulates the disordered gut microbiota, and mitigates metabolic endotoxemia and inflammation in type 2 diabetic rats. J. Endocrinol. Investig. 46 (4), 699–711. 10.1007/s40618-022-01914-3 36219316

[B137] Zhang X. Yuan S. Fan H. Zhang W. Zhang H. (2024). Liensinine alleviates sepsis-induced acute liver injury by inhibiting the NF-κB and MAPK pathways in an Nrf2-dependent manner. Chem. Biol. Interact. 396, 111030. 10.1016/j.cbi.2024.111030 38692452

[B138] Zhao J. Yu H. Liu Y. Gibson S. A. Yan Z. Xu X. (2016). Protective effect of suppressing STAT3 activity in LPS-Induced acute lung injury. Am. J. Physiol. Lung Cell. Mol. Physiol. 311 (5), L868–l880. 10.1152/ajplung.00281.2016 27638904 PMC5130536

[B139] Zhao H. Jiang Z. Chang X. Xue H. Yahefu W. Zhang X. (2018). 4-Hydroxyphenylacetic acid prevents acute APAP-induced liver injury by increasing phase II and antioxidant enzymes in mice. Front. Pharmacol. 9, 653. 10.3389/fphar.2018.00653 29973881 PMC6020787

[B140] Zhao J. Ming J. Hu X. Chen G. Liu J. Yang C. (2020). Bayesian weighted Mendelian randomization for causal inference based on summary statistics. Bioinformatics 36 (5), 1501–1508. 10.1093/bioinformatics/btz749 31593215

[B141] Zhou S. Liu L. Ye B. Xu Y. You Y. Zhu S. (2024). Gut microbial metabolism is linked to variations in circulating non-high density lipoprotein cholesterol. EBioMedicine 104, 105150. 10.1016/j.ebiom.2024.105150 38728837 PMC11090025

