# Correction: A systematic exploration of gut microbiota–driven blood metabolites in sepsis: an integrated bioinformatics and genetic association study

**DOI:** 10.3389/fgene.2026.1826365

**Published:** 2026-03-26

**Authors:** Yanhuo Zhang, Chenghao Qiu, Xingyu Li, Yaling Peng, Jun Liu, Peng Zhu

**Affiliations:** 1 Department of Gastrointestinal Surgery, The Second Affiliated Hospital of Chongqing Medical University, Chongqing, China; 2 The Second Clinical Medical College of Chongqing Medical University, Chongqing, China; 3 Department of Anesthesiology, Western Medicine Department, The Central Hospital of Enshi Tujia and Miao Autonomous Prefecture, Enshi, Hubei, China

**Keywords:** genome-wide association study, gut microbiota, metabolites, network pharmacology, sepsis

Incorrect funding.

The funder Chongqing Natural Science Foundation, CSTB2024NSCQ-KJFZMSX0101 to Peng Zhu was erroneously omitted.

The original article has been updated.

