# Supplementary material for: A systematic exploration of gut microbiota–driven blood metabolites in sepsis: an integrated bioinformatics and genetic association study
Source: Front Genet. 2026 Mar 2;17:1754817. doi: 10.3389/fgene.2026.1754817 (PMC12989214; doi:10.3389/fgene.2026.1754817)
Supplement: Supplementary file 2 [file DataSheet1.docx]

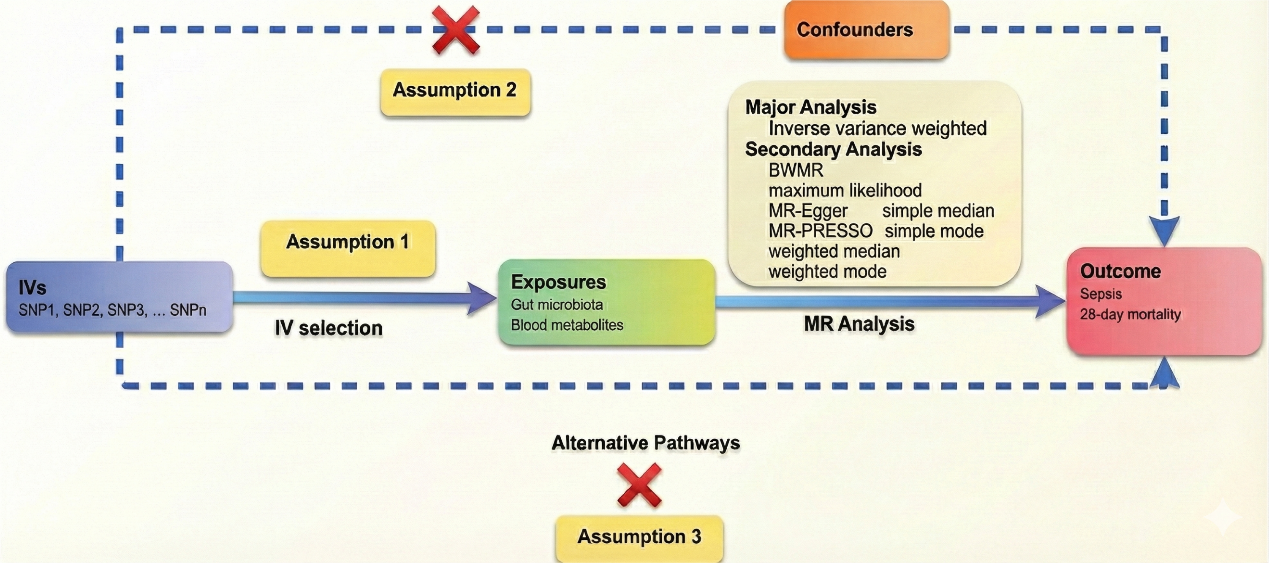


Fig. S1. Schematic diagram illustrating the classical MR framework designed for this study. Solid blue arrows denote hypothesized causal relations, pointing from the genetic instrumental variables (IVs) to the exposures, and subsequently to the outcomes. The red “X” marks indicate blocked pathways, representing the necessary assumptions for valid causal inference in MR (independence from confounders and absence of alternative pathways/horizontal pleiotropy). The dashed frame and lines represent potential confounding factors. MR, Mendelian randomization; IV, instrumental variable; SNP, single nucleotide polymorphism.
